# Mouse models of COVID-19 recapitulate inflammatory pathways rather than gene expression

**DOI:** 10.1371/journal.ppat.1010867

**Published:** 2022-09-26

**Authors:** Cameron R. Bishop, Troy Dumenil, Daniel J. Rawle, Thuy T. Le, Kexin Yan, Bing Tang, Gunter Hartel, Andreas Suhrbier

**Affiliations:** 1 Immunology Department, QIMR Berghofer Medical Research Institute, Brisbane, Queensland, Australia; 2 Statistics Unit, QIMR Berghofer Medical Research Institute, Brisbane, Queensland, Australia; 3 Australian Infectious Disease Research Centre, GVN Center of Excellence, Brisbane, Queensland, Australia; University of Iowa, UNITED STATES

## Abstract

How well mouse models recapitulate the transcriptional profiles seen in humans remains debatable, with both conservation and diversity identified in various settings. Herein we use RNA-Seq data and bioinformatics approaches to analyze the transcriptional responses in SARS-CoV-2 infected lungs, comparing 4 human studies with the widely used K18-hACE2 mouse model, a model where hACE2 is expressed from the mouse ACE2 promoter, and a model that uses a mouse adapted virus and wild-type mice. Overlap of single copy orthologue differentially expressed genes (scoDEGs) between human and mouse studies was generally poor (≈15–35%). Rather than being associated with batch, sample treatment, viral load, lung damage or mouse model, the poor overlaps were primarily due to scoDEG expression differences between species. Importantly, analyses of immune signatures and inflammatory pathways illustrated highly significant concordances between species. As immunity and immunopathology are the focus of most studies, these mouse models can thus be viewed as representative and relevant models of COVID-19.

## Author summary

Herein we used RNA-Seq data and bioinformatics to address how faithfully mouse models of COVID-19 recapitulate human transcriptional profiles. Significant gene expression changes seen during SARS-CoV-2 infection of mouse lungs in 3 mouse models did not show good overlap with significant gene expression changes seen during SARS-CoV-2 infection of human lungs. However, highly significant levels of concordance were seen between species when immune signatures and inflammatory pathways were compared. At the immune and inflammation pathway levels, the mouse models thus emerge as representative and relevant models of COVID-19.

## Introduction

Mouse models represent critical tools for preclinical evaluation of new interventions and for understanding disease, host responses and pathogen behaviors. However, views on how well mice recapitulate human transcriptional profiles range from substantial conservation [1,2] to considerable diversity [3]. In pro-inflammatory settings, reports have also argued that mouse models mimic human transcriptional responses well [4] or poorly [5]. Mouse models of human disease can thus be seen as less reliable [6–10] or, in other settings, as recapitulating faithfully key elements of human disease [11–13]. Given both conservation and diversity can be identified [14,15], specifically interrogating any given mouse model for how reliably its transcriptomic responses mimic those seen in humans may be warranted [16–18].

A widely used mouse model of SARS-CoV-2 infection and COVID-19 disease is the K18-hACE2 mouse, where the human angiotensin-converting enzyme 2 (hACE2) is expressed as a transgene from the keratin 18 (K18) promoter. These mice develop a robust respiratory disease that histologically resembles severe COVID-19 [19–21]. These mice have been widely used for evaluation of new interventions [22–29], and for virology and immunopathology studies [30–32]. However, SARS-CoV-2 infected K18-hACE2 mice show a number of differences [19], perhaps the most important difference is a fulminant brain infection that is associated with the generally lethal outcome in this model after infection with original strains of SARS-CoV-2 [33]. Brain infection can be avoided by using aerosol delivery [34] rather than inoculation into the lungs via the intranasal route, however, the latter has been more widely adopted and is used herein. Human brain infection has now been demonstrated [35], although fulminant lethal brain infection is not a feature of COVID-19 [36,37]. A second mouse model of SARS-CoV-2 infection and COVID-19 disease involves expression (also as a transgene) of hACE2 driven by the mouse ACE2 (mACE2) promoter [38, 39], which we independently generate herein and refer to as mACE2-hACE2 mice. This mouse model of COVID-19 is generally less severe, with infections usually self-limiting and non-lethal [38,39]. A third model used a mouse-adapted strain of an original SARS-CoV-2 isolate, MA1, which is able to utilize mACE2 and is able to infect wild-type mice efficiently [32]. Herein we elucidate bioinformatic methods for validating mouse models by comparing the transcriptional responses of mouse lungs and human lungs/lung tissues after SARS-CoV-2 infection. Although overlap of differentially expressed genes between human studies and all three mouse models was universally poor, concordance for immune signatures and inflammation pathways was high.

## Results

### K18-hACE2 mouse and human data sets for SARS-CoV-2 infected lung tissues

RNA-Seq data sets from lungs of SARS-CoV-2 infected K18-hACE2 mice were obtained from two independent sources, Winkler and our own group, Suhrbier (Table 1). For the former [40], datasets from lungs of SARS-CoV-2 infected K18-hACE2 mice for 2, 4 and 7 days post infection (dpi), were obtained from the NCBI Sequence Read Archive (SRA) (Table 1). Two Suhrbier K18-hACE2 datasets were available, 2 and 4 dpi, with 4 dpi data reported previously [27] and 2 dpi data generated for this study (Table 1). Fastq files were analyzed or reanalyzed herein using STAR, RSEM and EdgeR, with a q<0.05 filter applied to provide Differentially Expressed Genes (DEGs) (S1 File, sheets A, D, G, J, and M). For each of these DEG lists, a mouse-human orthologue DEG list (orthoDEGs) (S1 File, sheets B, E, H, K, and N) and a single copy orthologue DEG list (scoDEGs) was generated (S1 File, sheets C, F, I, L, and O).

**Table 1:** Origins of human and mouse gene expression datasets.

Study name	Species	Dataset source	Infected Tissue	Control Tissue	Method	Platform	Re-analyzed	Notes
Winkler 2, 4 & 7 dpi	Mouse (K18-hACE2)	PRJNA645133	SARS-CoV-2 infected lung	Mock infected lung	RNA-Seq (poly-A selected)	Illumina NovaSeq 6000	Y	2.5x10^4^ p.f.u. n = 4–6 per group
Suhrbier 2 & 4 dpi	Mouse (K18-hACE2)	PRJNA767499 PRJEB43658	SARS-CoV-2 infected lung	Mock infected lung	RNA-Seq (poly-A selected)	Illumina NextSeq 550	Y	5x10^4^ CCID_50_ n = 4–6 per group
mACE2-hACE2 2, 4, 6 & 10 dpi	Mouse (mACE2-hACE2)	PRJNA767499	SARS-CoV-2 infected lung	Mock infected lung	RNA-Seq (poly-A selected)	Illumina NextSeq 550	Y	5x10^4^ CCID_50_ n = 3/4 per group
Mouse adapted virus, MA1 4 dpi	Mouse C57BL/6J	PRJNA804321	SARS-CoV-2 infected lung	Mock infected lung	RNA-Seq (poly-A selected)	Illumina NextSeq 550	N	5x10^4^ CCID_50_ n = 5 per group
Wu	Human	PRJNA646224	Formalin fixed paraffin embedded post mortem COVID-19 lung	Formalin fixed paraffin embedded healthy tissue from lung cancer patients	RNA-Seq (rRNA-depleted)	Illumina NextSeq 550	Y	Infected n = 9, Control n = 10. Medicated.
Alfi	Human	PRJNA688321	SARS-Cov-2 infected *Ex vivo* 3D lung organ culture	mock infected *Ex vivo* 3D lung organ culture	RNA-Seq (poly-A selected)	Illumina NextSeq 500	Y	Infected n = 10 Control n = 10
Blanco-Melo	Human	PRJNA615032	Formalin fixed COVID-19 post-mortem lung	Healthy post-surgery lung biopsy	RNA-Seq (poly-A selected)	Illumina NextSeq 500	N	Infected n = 2 Control n = 2 4 RNA-Seq libraries for each.
Acker-mann	Human	Vivli Center for Clinical Research Data	Formalin fixed COVID-19 post-mortem lung	Formalin fixed healthy donated lung	Nano-String	nCounter Inflamma-tion Panel	N	Infected n = 7, Control n = 10 Alveolar damage noted

K18-hACE2 mouse studies from two independent laboratories provided five data sets from lungs of SARS-CoV-2-infected K18-hACE2 mice. K18-hACE2 data covered three time points, specifically, days post infection (dpi). Four mACE2-hACE2 datasets were generated from lungs of SARS-CoV-2-infected mACE2-hACE2 mice, and covered four time points. The previously published MA1 dataset was derived from lungs of C57BL/6J mice infected with the MA1 mouse-adapted strain of SARS-CoV-2. Four independent human studies provided three gene expression datasets from SARS-CoV-2-infected lungs, and one from 3D lung organ culture infected *ex vivo* with SARS-CoV-2. The Ackerman study analyzed expression of 249 mRNAs associated with inflammation. All infections were with SARS-CoV-2 isolates belonging to the original or ancestral lineage. PRJ prefixed annotations represent NCBI Bioproject accession numbers. Reanalysis means raw fastq files were re-analyzed for this study using STAR, RSEM and EdgeR.

Three RNA-Seq datasets derived from SARS-CoV-2 infected human lung samples (COVID-19 vs. Controls) were obtained from the NCBI Sequence Read Archive (SRA) and are referred to as Wu [41], Alfi [42] and Blanco-Melo [43] (Table 1). The Alfi and Wu data sets were reanalyzed (using STAR, RSEM and EdgeR) to produce gene expression datasets. Compared to the Alfi and Wu data sets, the Blanco-Melo dataset showed very low sequencing depth in the COVID-19 samples (S1 Fig), and the original gene list provided by the authors [43] was used. DEGs (S1 File, sheets P, S, and V), orthoDEGs (S1 File, sheets Q, T, and W) and scoDEGs (S1 File, sheets R, U, and X) were generated as above for the 3 human groups, respectively. An additional human COVID-19 DEG list derived from NanoString analysis [44] was obtained from the Vivli Center for Clinical Research Data (Table 1, Ackermann).

**Fig 1:**
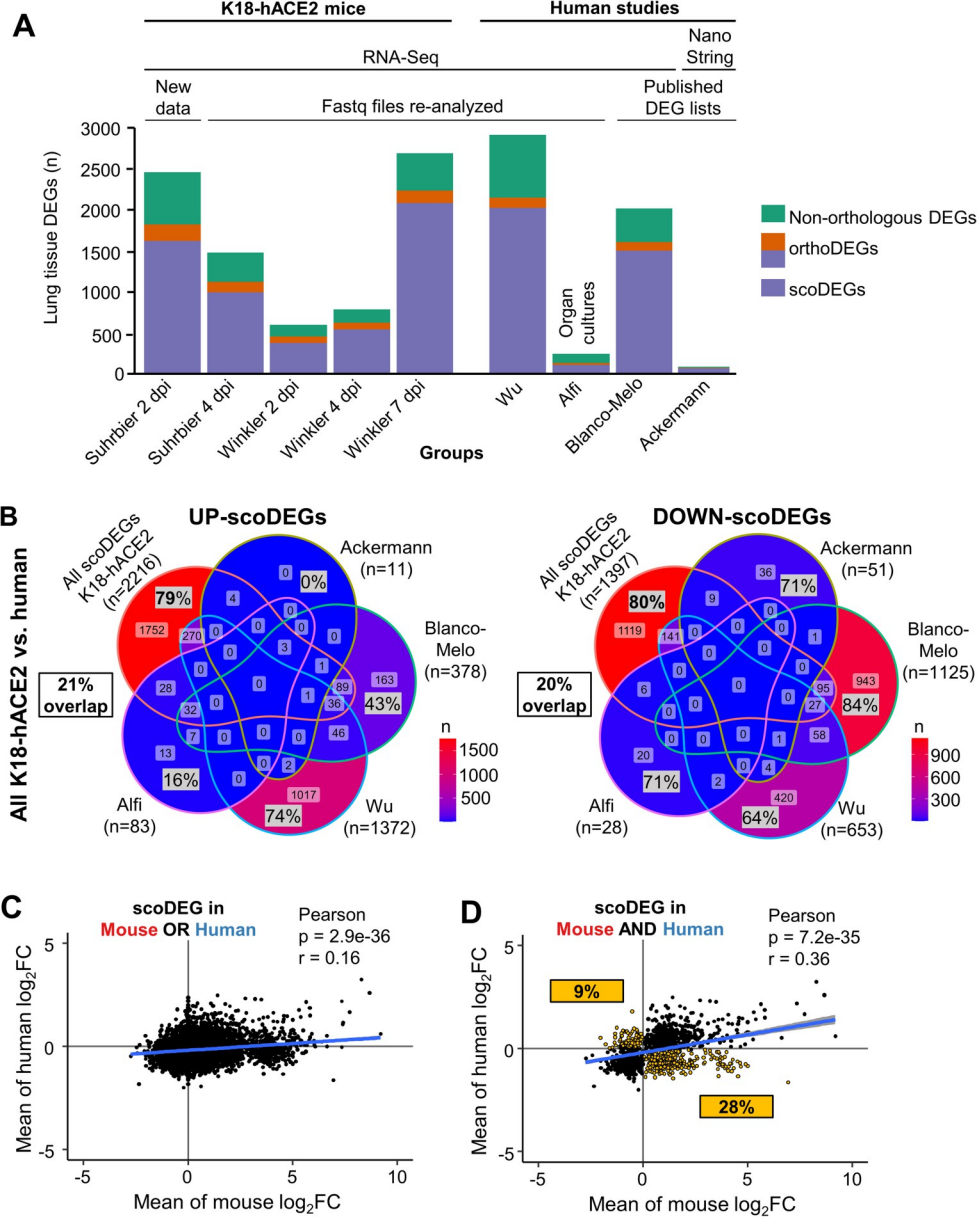
Mouse and human DEGs; overlaps and concordances between species. **(A)** DEGs from lungs/lung tissues infected with SARS-CoV-2 were identified in K18-hACE2 mouse and human studies (n = number of DEGs). DEGs were generated from original RNA-Seq data provided herein (New Data), re-analyzed from previously published RNA-Seq data (Fastq files re-analyzed), or were obtained from publications (published DEG lists). All datasets were derived from RNA-Seq, except Ackerman which was obtained from a Nanostring study. Coloring of bars: Green—non-orthologous between mouse and human; Orange—with one or both species having multiple orthologues; Purple—both species having a single copy orthologue. A total of 9 Groups (5 K18-hACE2 and 4 human) were considered in the subsequent analyses. **(B)** The union of all K18-hACE2 scoDEGs was used to compare mouse and human for up- and down-regulated scoDEGs. ‘n’ refers to the number of scoDEGs for each group. Percentages within the Venn diagram (gray boxes) show the percentage of scoDEGs exclusive to that group (i.e. a scoDEG in that group but no other group. E.g. 1752/2216 x 100 = 79%). The boxed overlap percentages represent the percentage of mouse scoDEGs that are also scoDEGs in one or more human studies (e.g. 2216-1752/2216 x 100 ≈ 21% for up-regulated scoDEGS and 1119-1397/1397 x100 ≈20% for down-regulated scoDEGs). **(C)** Pearson correlation of mean log_2_FCs of single-copy orthologues that were DEGs in either any mouse group or any human group or both. **(D)** Pearson correlation of mean log_2_FCs of single-copy orthologues that were DEGs in both one or more mouse groups and one or more human groups. scoDEGs that had inconsistent mean expression between species (i.e. were upregulated in one species and down-regulated in another) are shown yellow. The percentage of scoDEGs with inconsistent expression (yellow boxes) is provided relative to the total number of scoDEGs.

In summary, five DEG, orthoDEG and scoDEG datasets for 3 time points and from two independent groups, describe significant differential gene expression in lungs of SARS-CoV-2-infected K18-hACE2 mice. Four independent DEG, orthoDEG and scoDEG datasets were obtained that describe significant differential gene expression in SARS-CoV-2-infected human lung tissues. The number of DEGs ranged from 75 to 2794 per dataset (Fig 1A). The proportion of DEGs in each dataset that were orthoDEGs or scoDEGs between K18-hACE2 mice and human ranged from 57 to 92% and 49 to 83%, respectively (Fig 1A). When the NanoString (Ackerman) and the Organ culture (Alfi) were removed, these percentages were 74 to 83% and 63 to 77%, respectively (Fig 1A).

### Poor overlap between scoDEGs from human and K18-hACE2 mouse studies

When the up-regulated scoDEGs from all the K18-hACE2 mouse data sets were combined (n = 2216) and compared with scoDEGs from each of the four human studies, 79% of scoDEGs up-regulated in mice were not up-regulated in any human study (Fig 1B). The same comparison for down-regulated scoDEGs showed 80% of scoDEGs down-regulated in mice were not down-regulated in any human study (Fig 1B). Thus, the overall overlap for up- and down-regulated scoDEGs for K18-hACE2 mice and human studies was only 21 and 20%, respectively.

Conceivably, due to the q<0.05 cutoff, a DEG in one species may narrowly have missed being a DEG in the other species due to it narrowly missing out on significance. A K18-hACE2 mouse vs. human comparison was thus undertaken using single-copy orthologues that were differentially expressed in at least one mouse group OR at least one human group (union scoDEGs) (Fig 1C), and a second comparison using single-copy orthologues that were differentially expressed in at least one mouse group AND at least one human group (intersection scoDEGs) (Fig 1D). For each comparison, log_2_ fold-changes (log_2_FC) were averaged within each species (S2 File) and tested for correlation. Although significant, correlations were relatively poor in both comparisons; r = 0.16 and 0.36 for union scoDEGs and intersection scoDEGs, respectively (Fig 1C and 1D). The poor overlap between scoDEGs in K18-hACE2 mice and human studies cannot therefore be readily explained by genes narrowly missing the q<0.05 cutoff. In addition, 37% (9% plus 28%) of genes showed opposite directions of average fold change for mouse and human scoDEGs (Fig 1D, yellow boxes).

When scoDEG overlaps were calculated in pairwise comparisons between each group, overlaps between mouse and human data sets remained low, ranging from 1–9% (S2 Fig). This illustrated that no human group showed good concordance with any mouse group, and that overlaps were higher when multiple studies were combined (Fig 1B). These analyses illustrate that scoDEGs identified in SARS-CoV-2-infected lungs from K18-hACE2 mice and humans show a low level of overlap.

### Large variations in viral read counts for human and K18-hACE2 studies

**Fig 2:**
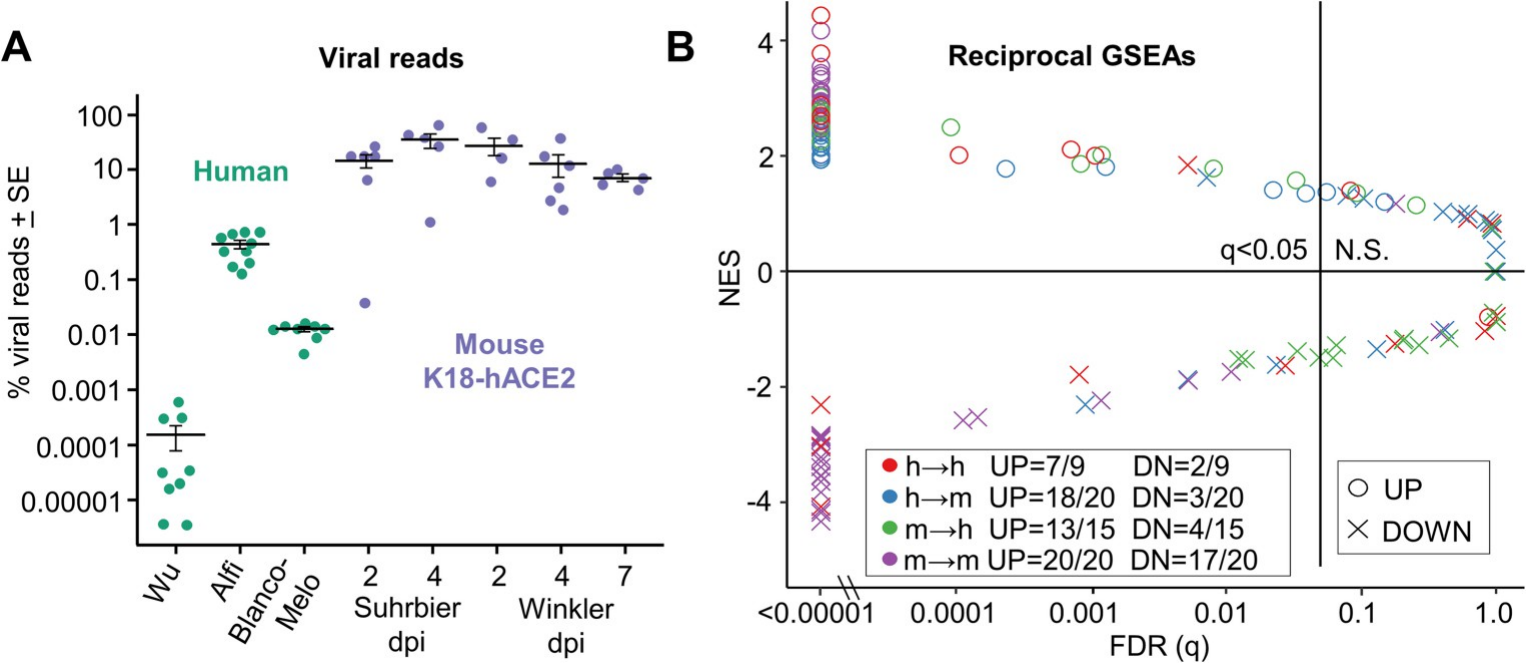
Viral reads and reciprocal GSEAs. **(A)** For each sample, the number of reads aligned to the SARS-CoV-2 genome are shown as a percentage of the total number of reads that align to all protein coding genes (filled circles). Cross-bars represent the mean for each group. **(B)** Pairwise reciprocal GSEAs showing enrichment of up- or down-regulated orthoDEG sets in log_2_FC ranked gene lists for all possible pair-wise comparisons between groups (i.e. 128 combinations; 3 human and 5 mouse ranked gene lists vs. 4 human and 5 mouse orthoDEG lists). Circles and crosses are colored according to direction of the GSEA (e.g. green = mouse orthoDEG sets vs. ranked human gene lists). Fractions show the proportion of orthoDEG sets that are significantly enriched with consistent directionality (e.g. “UP = 13/15” indicates that, of 15 GSEAs using up-regulated orthoDEG sets, 13 showed significant enrichment with positive NES).

To assess the viral loads for each group, the percentage of reads mapping to the virus was determined for each RNA-Seq dataset and expressed as a percentage of reads aligned to protein-coding genes. K18-hACE2 mouse groups had ≈1.5–5.5 logs more mean percent viral read counts than the human studies (Fig 2A). The high viral read counts in K18-hACE2 mice was perhaps not surprising given that this is a robust lethal model of SARS-CoV-2 infection [20, 30, 40].

The analysis also illustrated that the human studies showed large differences in viral read counts. Alfi contained ≈2 logs more percent viral reads than Blanco-Melo, and Blanco-Melo had ≈2 logs more percent viral reads than Wu. In contrast, the mean percent viral read counts varied by only ≈1 log between K18-hACE2 mouse groups (Fig 2A). The role of viral loads in lack of scoDEG concordance between species is analyzed further below.

### Reciprocal GSEAs show species concordance for upregulated orthoDEGs

A method for comparing gene expression data sets is to use Gene Set Enrichment Analysis (GSEA), whereby the enrichment of a DEG set within a pre-ranked gene list is evaluated [45–48]. For orthologues with different gene nomenclature in mice and humans, the mouse gene symbols were changed to their orthologous human equivalent in the orthoDEG sets and the gene lists. This allows GSEAs to be undertaken for mouse vs. human gene sets. The orthoDEG gene sets used in the GSEAs comprised the top 50% of orthoDEGs ranked by fold change (S1 File). These orthoDEG sets were then used to interrogate ranked gene lists (ranked by fold change) for all the other groups (S4 File).

The up-regulated orthoDEG sets from almost all groups were significantly enriched with positive normalized enrichment scores (NES) in the ranked gene lists (Fig 2B, circles; and S5 File). Of the 35 (20 plus 15) GSEAs for mouse-human and human-mouse comparisons, 31 (18 plus 13) reached significance (Fig 2B, q<0.05 blue and green circles). Thus, although overlap in up-regulated orthoDEGs was poor and variation in viral loads was high, the top up-regulated orthoDEGs identified in SARS-CoV-2 infected lungs from K18-hACE2 mice generally showed significant enrichment by GSEAs in human ranked gene lists and *vice versa*.

GSEAs using down-regulated orthoDEGs provided the opposite result for mouse-human and human-mouse comparisons with only 7 (3 plus 4) out of 35 GSEAs reaching significance (Fig 2B, q<0.05, blue and green crosses; and S5 File). In addition, only 2/9 human-human GSEAs of down-regulated orthoDEGs reached significance (Fig 2B, red crosses). However, for mouse-mouse GSEAs, the number that reached significance for down-regulated orthoDEGs (17/20) was only marginally lower than for up-regulated orthoDEGs (20/20) (Fig 2B).

To gain insights into why the down-regulated orthoDEGs often did not reach significance in the GSEAs for mouse-human, human-mouse and human-human comparisons, the down-regulated DEGs were analyzed by Ingenuity Pathway Analysis (IPA) Diseases and Functions feature. Strikingly, when down-regulated DEGs were analyzed, the same top scoring annotation by z-score for all groups was Organismal death, with the exception of the Alfi organoid study, where the top annotation was Perinatal death (S6 File). Down-regulated DEG lists thus consistently contain tissue damage signatures, with cell death and injury in SARS-CoV-2 infected lungs induced via direct viral cytopathic effects [49] and via the cytokine storm [50]. Nevertheless, the very different levels of infection (and ensuing differences in tissue injury) may contribute to the aforementioned differences in down-regulated genes, with the comparable levels of virus infection seen in the K18-hACE2 mouse groups (Fig 2A) contributing to the higher level of congruence (i.e. 17/20 significant GSEAs) (Fig 2B, purple crosses).

### GSEAs using ImmuneSigDB show concordance between K18-hACE2 and human studies

**Fig 3:**
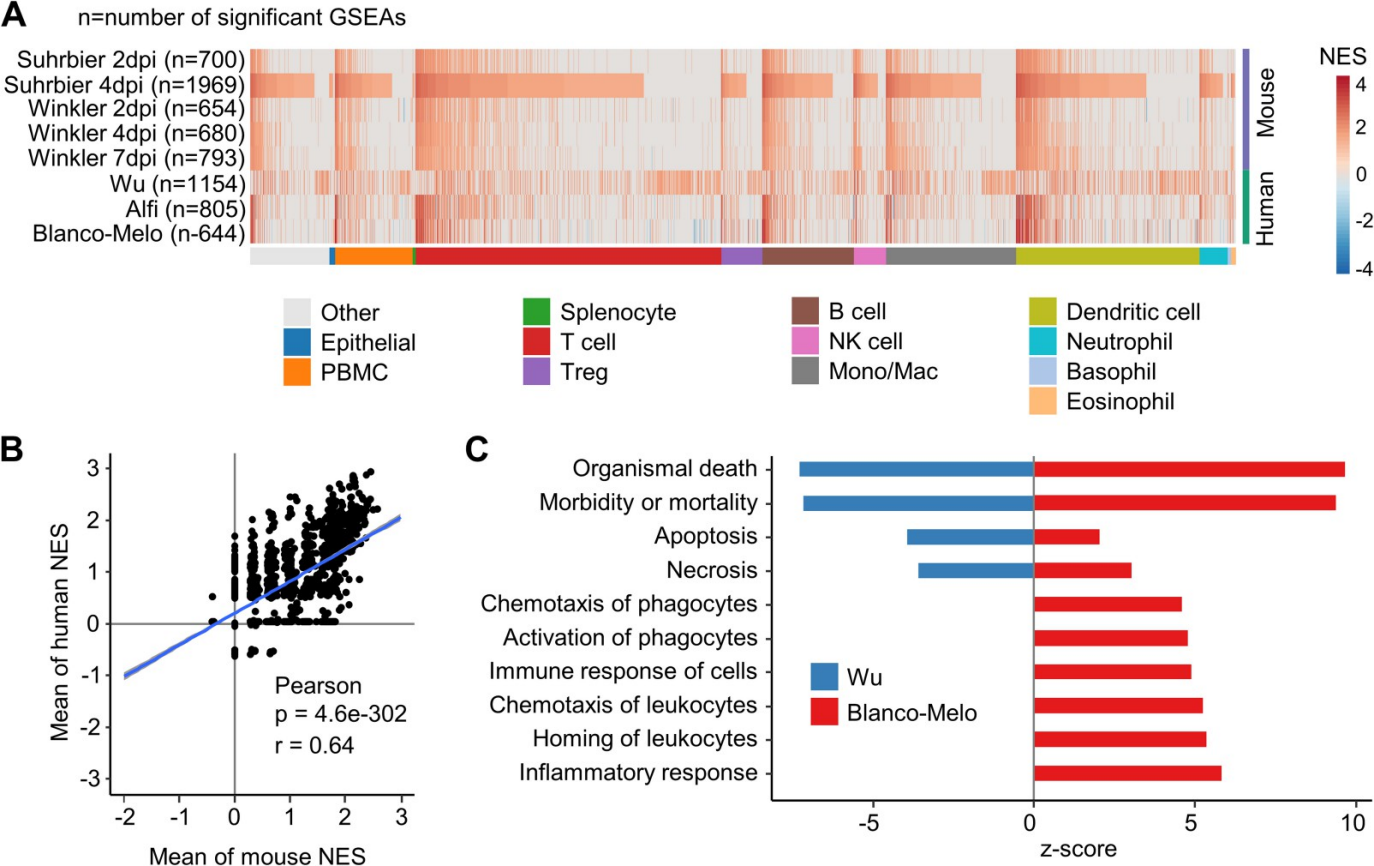
Gene Set Enrichment Analysis using immune-cell gene sets. **(A)** Gene sets from the GSEA Immune Signatures Database (ImmuneSigDB) were used to interrogate log2FC ranked gene lists from all groups. A total of 2879 GSEAs were significantly enriched in at least one group, and were clustered according to the cell-type mentioned in the annotation for the gene set. Within each cell-type cluster, gene sets were ranked according NES for Suhrbier 4 dpi. **(B)** Pearson’s correlation of mean mouse NES for 2879 ImmuneSigDB gene sets vs. the mean human NES for the same gene sets. **(C)** The full DEG lists for Wu and Blanco-Melo were analyzed by IPA Diseases and Functions. The top 10 annotations that had the greatest differences in z-scores between Wu and Blanco-Melo are shown, ranked by difference. A z-score of 0 means the annotation was not identified as significant by IPA.

ImmuneSigDB is a compendium of ≈5000 immunology-specific gene sets that can be used to interrogate mouse and human ranked gene lists using GSEAs to identify immune signatures [15]. GSEAs using ImmuneSigDB gene sets were used to interrogate the K18-hACE2 and human gene lists ranked by fold change (S4 File). The NES are plotted for GSEAs that reached significance (q<0.05), with the NES ranked by Suhrbier 4 dpi (Fig 3A), as this dataset provided the largest number of significant GSEA results (n = 1969). The results were also grouped by ImmuneSigDB gene sets that mentioned a specific cell type (Fig 3A) in the gene set annotation [15]. Concordance between the K18-hACE2 mouse and human GSEA results was generally high, with the concordance also apparent across all cell types (Fig 3A).

A Pearson correlation of the mean NES for human vs. K18-hACE2 mice provided a highly significant correlation (Fig 3B, r = 0.64), illustrating that both human and K18-hACE2 mouse datasets showed comparable enrichments for many ImmuneSigDB gene sets. In summary, despite the poor overlap for scoDEGs, GSEAs using ImmuneSigDB gene sets argue that human and K18-hACE2 mouse ranked gene lists share a significant number of immune related signatures.

The Wu dataset showed a clearly distinct pattern (Fig 3A). To gain insights into the reason for this, the DEGs (up and down-regulated) from the Wu and Blanco-Melo studies were analyzed by IPA Diseases and Functions. The results were ranked by difference in z-scores, with the top 10 most different annotations shown (Fig 3C). Cell death and inflammation annotations had much lower z-scores in the Wu study when compared with the Blanco-Melo study (Fig 3C). These results are consistent with the ≈2 log lower mean level of virus in the Wu group (Fig 2A), with methylprednisolone treatments in 4/9 patients (and interferon in one patient and intravenous immunoglobulin in another) [41] perhaps also suppressing inflammation and thereby prolonging survival [51] to a time when viral titers have waned. The antiviral treatments used in this study [41] have subsequently been shown not to have significant activity.

### Cytokine/chemokine signaling pathways show high concordance between human and K18-hACE2 studies

**Fig 4:**
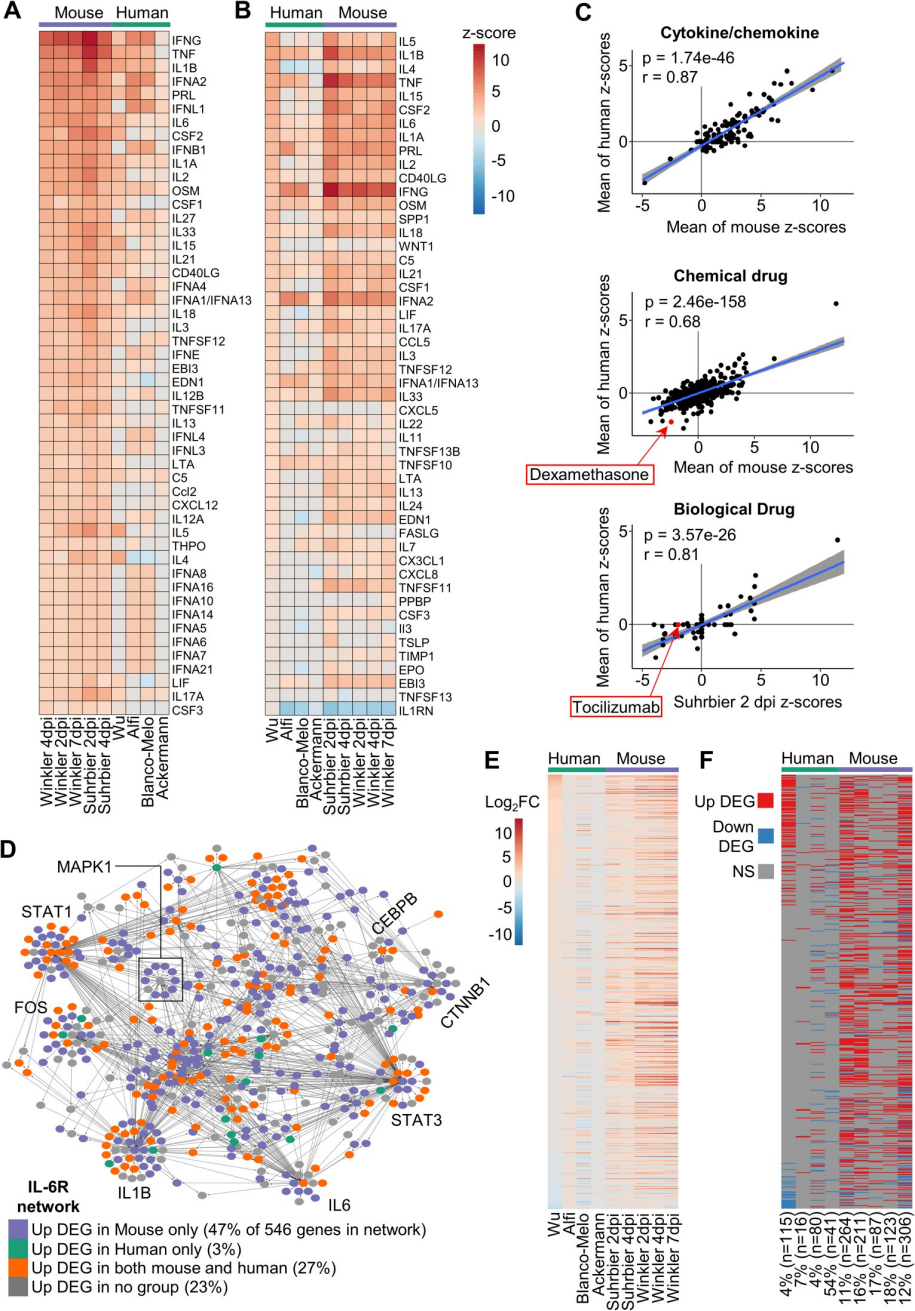
Cytokine/chemokine and drug IPA USR concordances between human and K18-hACE2 mice. **(A)** DEGs from each group were analyzed by the upstream regulator (USR) feature of IPA. The heatmap shows the top 50 of cytokine/chemokine USRs ranked by activation z-scores from the Winkler 4 dpi data. **(B)** Heatmap comparing groups as in A, except ranked according to z-score from the Wu data. **(C)** Cytokine/chemokine–Pearson correlation of mean mouse z-scores vs. mean human z-scores for significant Cytokine/chemokine USRs (n = 146). Chemical drugs–Pearson correlation of mean mouse z-scores vs. mean human z-scores for significant Chemical drug USRs (n = 1156). Biologic drugs—Pearson correlation of mean mouse z-scores vs. mean human z-scores for significant chemical drug USRs (n = 109). For calculating means, non-significant USRs were given a value of zero, thus means were derived from n = 5 for mouse groups and n = 4 for human groups. **(D)** Network of 546 genes associated with IL-6R signaling according to IPA. Node color indicates whether a gene was up-regulated only in mouse (purple; ≥1 mouse group, and no human group), only in human (green; ≥1 human group, and no mouse group), both (orange; ≥1 mouse and ≥1 human group), or not up-regulated in any group (grey). Large sub-networks are labeled according to their hub node. **(E)** Heatmap comparing groups according to log_2_ fold-change (log_2_FC) of 546 genes associated with the IL-6R signaling network. Genes are ranked according to log_2_FC in Wu. **(F)** Categorical “heatmap” with groups (x axis) and genes (y axis) as in E, with up-regulated DEGs shown in red, down-regulated DEGs in blue, and genes whose expression was not significantly different (NS) in grey. The percentage and number of DEGs in each group that are present in the IL-6R network is indicated; e.g. The Wu data set has 2875 DEGs, of which 4% (115) are present in the IL6R network.

For COVID-19 a key feature is up-regulation of inflammatory mediators, with the ensuing cytokine storm associated with acute respiratory distress syndrome (ARDS) that characterizes severe disease [52]. The 5 K18-hACE2 mouse and 4 human DEG sets were analyzed by IPA, which accepts both human and mouse gene nomenclatures. The Up-Stream Regulator (USR) feature of IPA provided a list of z-scores for cytokine (which includes chemokine) pathways, which were then ranked and plotted in heat maps. This analysis illustrated considerable concordance between the dominant pro-inflammatory cytokine/chemokine responses in K18-hACE2 mice and humans (Fig 4A and 4B). A highly significant correlation emerged between the mean z-scores for cytokine/chemokine USRs for human groups and the mean z-scores for cytokine/chemokine USRs for K18-hACE2 mouse groups (Fig 4C, Cytokine/chemokine). This correlation remained highly significant when cytokine/chemokine USR z-scores from individual K18-hACE2 data sets were used instead of the means (S4 Fig). Thus, although orthoDEGs and scoDEGs showed poor overlaps for human and K18-hACE2 groups, pathway analyses illustrated that the cytokine/chemokine responses in SARS-infected human and K18-hACE2 lung tissues were actually quite similar.

A series of human clinical trials have shown the benefit of anti-inflammatory treatments for COVID-19 ARDS such as corticosteroids (e.g. dexamethasone) and the anti-IL-6-receptor (tocilizumab) [53–55]. When IPA Chemical Drug USRs were compared, a highly significant correlation emerged, with dexamethasone appearing with the expected negative z-score in both mice and human data sets (Fig 4C, Chemical drug). When IPA Biologic Drug USRs were compared, a highly significant correlation again emerged, although only Suhrbier 2 and 4 dpi showed a negative z-score for tocilizumab (Fig 4C, Biological drug; and S7 File). Why the human data sets all failed to provide a z-score for tocilizumab (S7 File) is unclear, given the strong IL-6 signatures (Fig 4A and 4B). Conceivably, the human lung samples were collected too late, with the best results for tocilizumab treatment achieved when the drug was given early in infection [56]. Overall, these results argue that K18-hACE2 mice represent a suitable model in which to test biologics and chemotherapeutics for COVID-19.

### Analysis of dominant cytokine pathways shows high species concordance

For human and K18-hACE2 mouse comparisons, differential gene expression showed poor overlaps (Fig 1B and 1C), whereas pathway analyses showed highly significant correlations (Figs 3A and 3B and 4A–4C). To dissect this apparent incongruity, the IL-6 receptor signaling network (IL-6R network) was examined in detail. IL-6 was consistently identified as an USR with positive z-scores (Fig 4A and 4B), with excessive IL-6 levels also associated with COVID-19 ARDS [54]. An IL-6R network, comprising 546 genes, was generated in IPA (S8 File, sheet A). Each gene (node) was then colored depending on whether it was an up-regulated DEG in one or more mouse data sets (47% of the 546 genes in the network), an up-regulated DEG in one or more human data sets (3%), an up-regulated DEG in any human and any K18-hACE2 mouse data set (27%), or not an up-regulated DEG in any human or mouse data set (23%) (Fig 4D and S9 File, sheet A). A heat map of log_2_ fold change was generated for the 546 genes in the IL-6R network (Fig 4E) and a parallel map was generated to indicate which genes were DEGs (q<0.05) for each data set (Fig 4F). Differences in gene expression patterns were again evident for human and K18-hACE2 mouse comparisons, even for genes within this dominant pathway (Fig 4E and 4F). This analysis was repeated for TNF and IFNg networks (also identified as dominant USRs, Fig 4A and 4B), with broadly similar results (S8 File, sheet B and C; S9 File, sheet B and C; S5 and S6 Figs). These results again illustrated that the percentage of genes significantly up-regulated in both human and mouse was relatively low (range 20–27%), even within these dominant pathways. These analyses also showed no evidence of species-specific clustering in the networks, with DEGs from both species spread across the networks and sub-networks (Figs 4D, S5 and S6).

That some genes were significantly up-regulated in K18-hACE2 mice, but not in humans, may, in part (see below), be explained by the substantially higher levels of virus infection in mice (Fig 2A). However, a number of genes were up-regulated DEGs in humans, but not in K18-hACE2 mice (18% for TNF, S5 Fig; 21% for IFNg, S6 Fig, green); also seen to some extent for the IL-6R network (3%, Fig 4D). (This was not primarily because these human DEGs had no orthologues in mice, as this accounted for only 4 to 5% of these genes). Conceivably, humans may up-regulate genes, even within these dominant networks, that mice do not. However, it emerged that this result was largely due to the Wu dataset (S7 Fig), which was already shown to have some unique features (Figs 2A, 3A and 3C). The use of medication in the Wu study, but not in any mouse study, may, for instance, explain why some genes within these dominant networks emerge to be up-regulated in humans, but not in mice, in our analyses (Figs 4D, S5 and S6, green).

Taken together these analyses argue that, although transcriptomic responses may show poor scoDEG overlap between K18-hACE2 mice and human studies for various reasons, they nevertheless, often indicate the activation of common pathways.

### Differences in K18-hACE2 backgrounds for Winkler and Suhrbier studies

Perhaps surprising was that the Winkler and Suhrbier datasets did not show a higher level of DEG overlap (Figs 4E, 4F and S3B). IPA Diseases and Functions analyses for lungs 2 dpi (where mean viral loads were similar, Fig 2A) also showed that cellular infiltrate and immune activation annotations had higher z-scores for the Suhrbier group than for the Winkler group (Table 2). The basis for these differences was unclear given both studies ostensibly used the same inbred K18-hACE2 mice supplied by The Jackson Laboratories, and both were infected with virus isolates belonging to the original SARS-CoV-2 strain (Table 1).

**Table 2:** IPA ‘Diseases and Functions’ differences between Suhrbier 2 dpi vs. Winkler 2 dpi.

Diseases and Functions	Suhrbier z-score	Winkler z-score	Delta z
Homing of leukocytes	5.937	0	5.937
Recruitment of blood cells	5.72	0	5.72
Phagocytosis of leukocytes	5.499	0	5.499
Metabolism of reactive oxygen species	5.33	0	5.33
Homing of granulocytes	5.228	0	5.228
Chemotaxis of granulocytes	5.228	0	5.228
Stimulation of cells	4.991	0	4.991
Homing of neutrophils	4.827	0	4.827
Maturation of blood cells	4.719	0	4.719
Migration of lymphatic system cells	4.606	0	4.606
Endocytosis by eukaryotic cells	4.582	0	4.582
Cell viability of blood cells	4.518	0	4.518
Cytotoxicity of cells	4.451	0	4.451
Activation of lymphatic system cells	4.308	0	4.308
Cell viability of leukocytes	4.276	0	4.276
Activation of granulocytes	4.116	0	4.116
Differentiation of T lymphocytes	4.069	0	4.069
Inhibition of blood cells	4.057	0	4.057
Binding of endothelial cells	4.02	0	4.02
Response of lymphocytes	4.016	0	4.016
Adhesion of immune cells	6.73	2.722	4.008
Recruitment of lymphocytes	3.98	0	3.98
Adhesion of blood cells	6.635	2.66	3.975
Replication of Flaviviridae	0	-3.959	3.959
Binding of blood cells	6.836	2.888	3.948
Homeostasis of leukocytes	3.865	0	3.865
Cell movement of lymphatic system cells	3.849	0	3.849
Inhibition of cells	3.83	0	3.83
Degranulation	3.826	0	3.826

DEGs for Suhrbier 2 dpi vs. Winkler 2 dpi. were separately analyzed by Diseases and Functions feature of IPA. Diseases and Functions were ranked by the difference in z-scores (Delta z), with the top 30 with the largest differences shown. Any Diseases and Functions that were not significant were given a z-score of 0. Diseases and Functions associated with leukocyte migration are highlighted in grey. On 2 dpi viral loads in lungs were comparable for Suhrbier and Winkler.

**Fig 5:**
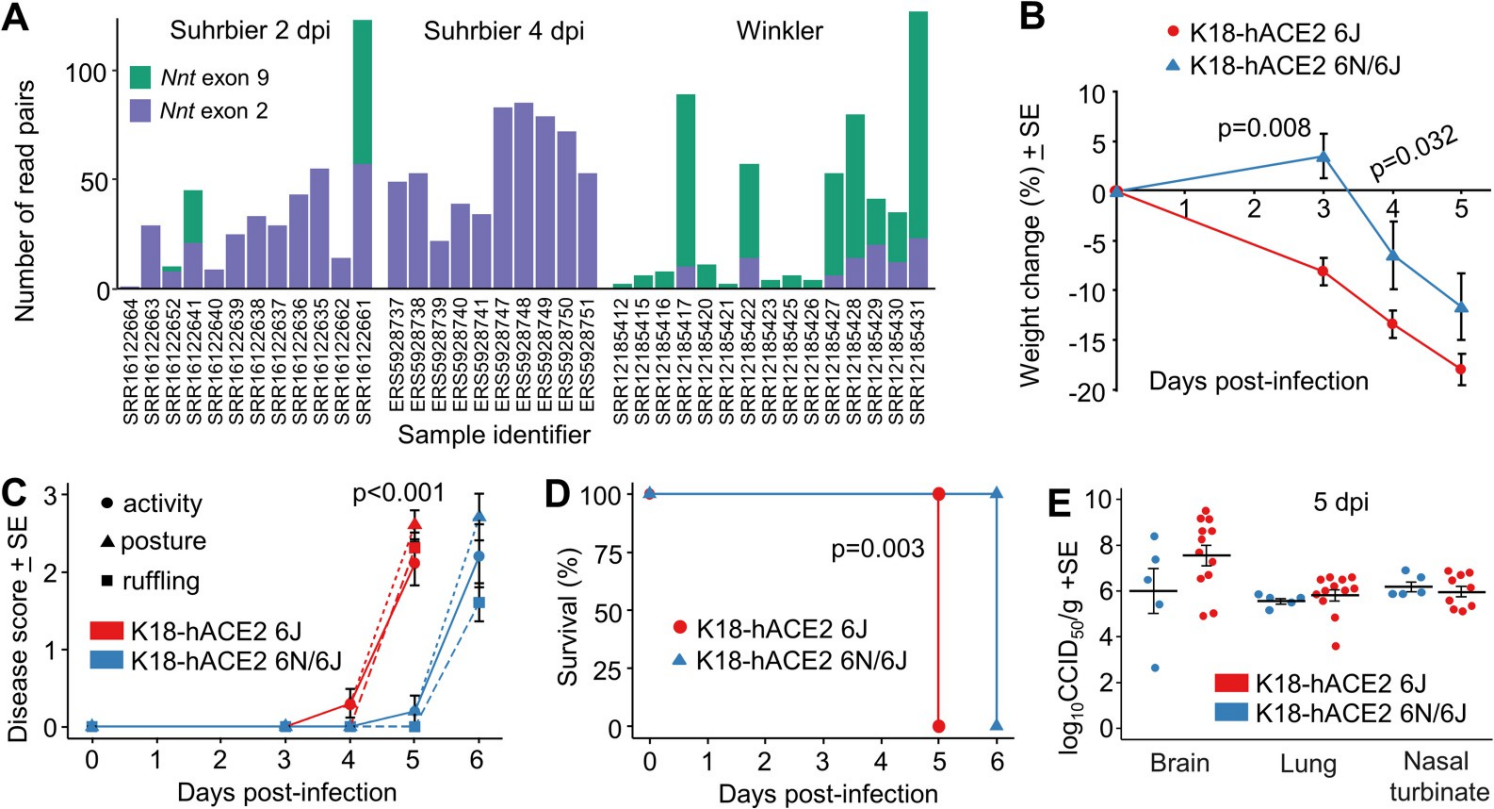
Genetic background of K18-hACE2 mice affects disease progression. **(A)** For each sample of the Suhrbier and Winkler datasets, the number of read pairs aligning to exon 9 and exon 2 of the *Nnt* gene are shown as green and purple bars, respectively. *Nnt* exon 9 is deleted in C57BL/6J mice. **(B)** Change in body weight over five days following SARS-CoV-2 infection is shown as a percentage of starting body weight for K18-hACE2 6J (*Nnt*^-/-^) and K18-hACE2 6J/6N (*Nnt* ^-/+^) mice; p-values indicate significant difference between means at 3 and 4 dpi (Mann Whitney U tests, n = 5 per group). **(C)** Three disease score parameters (activity, posture and fur ruffling) over six days following SARS-CoV-2 infection are shown for K18-hACE2 6J and K18-hACE2 6J/6N mice. Statistics for 5 dpi for all 3 parameters by Kolmogorov-Smirnov tests (n = 5 per group). **(D)** Kaplan-Meier curves showing survival for K18-hACE2 6J and K18-hACE2 6J/6N mice following SARS-CoV-2 infection. Significance by log rank test (n = 5 per group). **(E)** Log_10_CCID_50_/g in brain, lung and nasal turbinate for K18-hACE2 6J and K18-hACE2 6J/6N mice 5 dpi with SARS-CoV-2. 6J data was derived from 2 independent experiments. Differences between K18-hACE2 6J and K18-hACE2 6J/6N mice for any tissue were not significance.

The K18-hACE2 founder line was created on a mixed C57BL/6J x SJL/J background [57]. Perhaps under-appreciated is that C57BL/6J (6J) mice contain a unique loss-of-function deletion of exons 5 to 9 of the *Nicotinamide nucleotide transhydrogenase* (*Nnt* gene), whereas most mouse strains (including SJL/J mice) encode a full length *Nnt* gene [58]. An exact-match k-mer method targeting exon 2 and 9 of the *Nnt* gene was used to interrogate the RNA-Seq data from the Suhrbier and Winkler studies. Most mice from the Suhrbier data sets had no exon 9 reads (Fig 5A), consistent with a dominant 6J background, with these mice maintained in-house as heterozygotes by repeated backcrossing onto 6J mice. In contrast, all of the K18-hACE2 mice from the Winkler study had exon 9 reads, arguing that these mice had an intact *Nnt* gene and that this line had not been extensively backcrossed onto 6J.

To explore further the influence of genetic background, we undertook a single back-cross of our K18-hACE2 mice (6J background, *Nnt*^*-/-*^) with C57BL/6N (6N) mice (*Nnt*^*+/+*^), and compared SARS-CoV-2 infection in (i) K18-hACE2 mice on 6J background (K18-hACE2 6J, *Nnt*^*-/-*^) with (ii) K18-hACE2 mice on a mixed 6N x 6J background (K18-hACE2 6N/6J, *Nnt*^*+/-*^). Weight loss, disease scores and survival were all significantly worse in K18-hACE2 6J mice than in the K18-hACE2 6N/6J mice (Fig 5B and 5D). The genetic background can thus influence SARS-CoV-2 induced inflammatory immunopathology in the K18-hACE2 model (Table 2); an observation consistent with a previous report that showed such a mixed background can also significantly affect an arthritic inflammatory immunopathology [58]. The *Nnt* gene may, at least in part, be responsible as NNT controls mitochondrial reactive oxygen species via the glutathione and thioredoxin pathways, and thus generally exerts an anti-inflammatory influence [58]. No significant differences in tissue titres were observed (Fig 5E), consistent with previous studies which also showed no detectable influence of such a mixed background on antiviral activity against an alphavirus [58].

In summary, the differences seen for the Winkler vs. Suhrbier data sets can be explained by the differences in the genetic backgrounds of the K18-hACE2 mice.

### The mACE2-hACE2 mouse model

**Fig 6:**
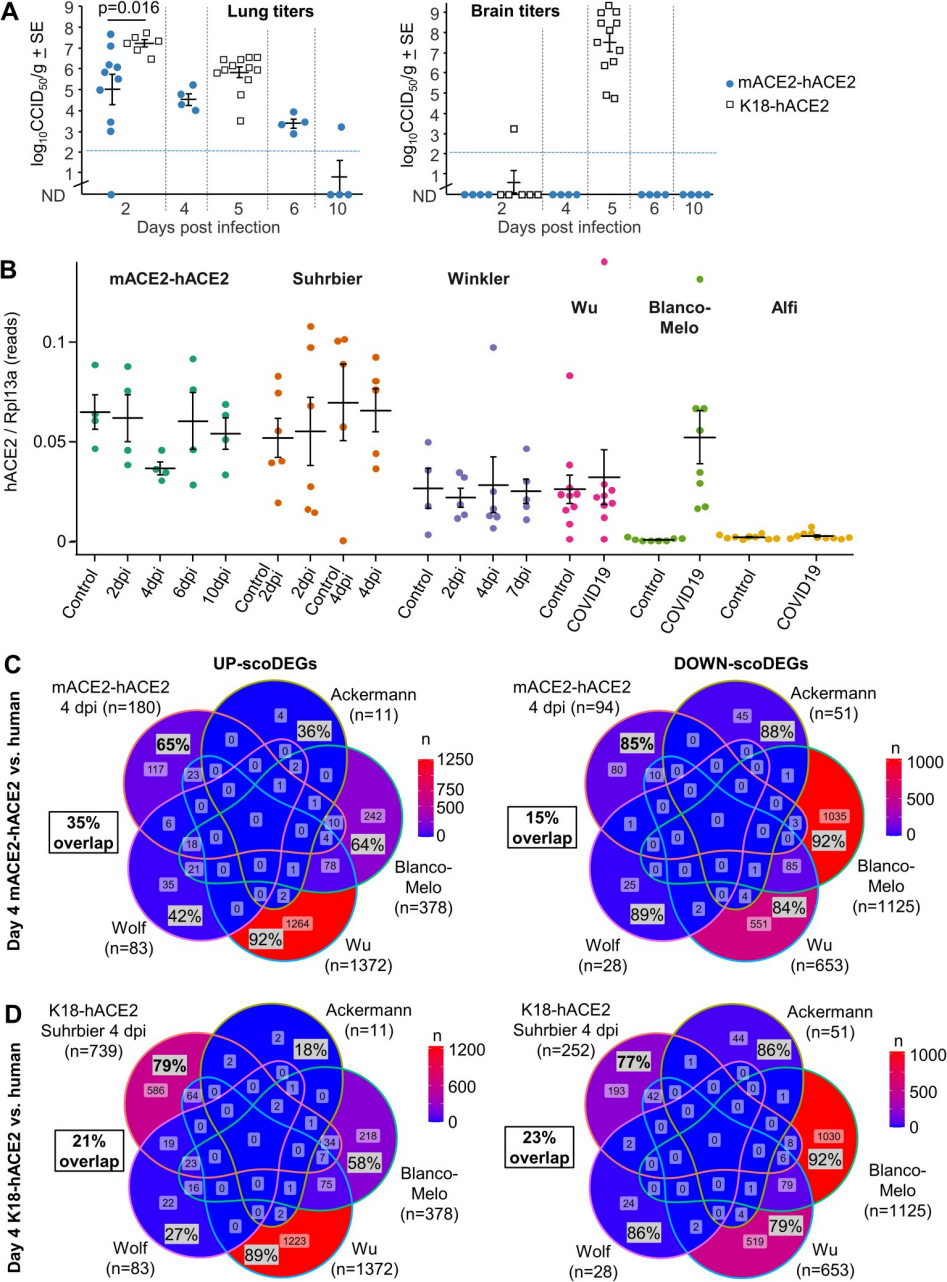
**The mACE2-hACE2 mouse model (A)** Lung and brain viral tissue titers at the indicated days post infection. All infected K18-hACE2 mice reach ethically defined endpoints for euthanasia (weight loss ≥20%) by day 5. None of the mACE-hACE2 mice reach ethically defined endpoints for euthanasia. Mean lung titers on day 2 were 2.2 log_10_CCID_50_ lower in mACE2-hACE2 mice compared to (Suhrbier) K18-hACE2 mice (p = 0.016, Kolmogorov-Smirnov test). (**B)** hACE2 reads normalized to Rpl13a reads for all mACE2-hACE2, K18-hACE2 and human lung samples. Cross-bars represent group means ± standard error. **(C)** Venn-diagrams show overlap in up- and down-regulated scoDEGs between mACE2-hACE2 4 dpi mice and the four human groups. Percentages within the Venn diagram (grey boxes) show the percentage of scoDEGs exclusive to that group (i.e. a scoDEG in that group, but in no other group). The boxed overlap percentages represent the percentage of 4 dpi mouse scoDEGs that are also scoDEGs in one or more human studies (e.g. 180-117/180 x 100 ≈ 35% for up-regulated scoDEGS and 94-80/94 x100 ≈15% for down-regulated scoDEGs). **(D)** As for C showing overlaps in up- and down-regulated scoDEGs between K18-hACE2 Suhrbier 4 dpi and the four human groups. Percentages as in C.

A criticism of the K18-hACE2 mouse model has been that hACE2 expression is driven by the keratin 18 promoter, which results in a fulminant brain infection that is associated with mortality [33]. An alternative, less severe, non-lethal model of SARS-CoV-2 infection involves use of transgenic mice where expression of hACE2 is driven by the mACE2 promoter (mACE2-hACE2 mice) [38]. We independently generated this mouse model (S8A and S8B Fig) and show that lung titers in mACE2-hACE2 mice were lower than those seen in K18-hACE2 mice on day 2 post infection (≈2 logs) (Fig 6A). Weight loss was also less prominent reaching ≈ 7% by day 8, with K18-hACE2 mice approaching 20% by day 5 (S8C Fig). Although nasal turbinates are infected (S8D Fig) no detectable brain infections were seen in mACE2-hACE2 mice (Fig 6A). Lung histology shows characteristic loss of alveolar spaces, cellular infiltrates, smooth muscle hypertrophy/hyperplasia, and bronchial sloughing (S8E Fig), although this lung pathology is less severe than that seen in K18-hACE2 mice [30]. Our mACE2-hACE2 mice thus behave similarly to those described by Bao et al. 2020 [38].

### RNA-Seq of infected mACE2-hACE2 mouse lungs

RNA-Seq analyses of SARS-CoV-2 infected mACE2-hACE2 mice was undertaken for days 0, 2, 4, 6 and 10 post infection (Table 1 and S1 Fig). Expression levels of hACE2 mRNA in this model were not significantly different from those seen in K18-hACE2 mice (Fig 6B, mACE2-hACE2 and Suhrbier). The reduced viral loads 2 dpi in mACE2-hACE2 mouse lungs (Fig 6A) and lower pathogenicity was thus not due to overall lower levels of receptor expression in lungs (Fig 6B). hACE2 levels in the other groups (Fig 6B) may not be strictly comparable as *inter alia* the Winkler study sequenced total RNA, the Wu study sequenced ribo-depleted RNA (rather than poly adenylated RNA), the Blanco-Melo study had low read depth (S1 Fig), and the Alfi data was derived from organoids (Table 1).

The RNA-Seq analysis of infected mACE2-hACE2 mouse lungs provided a series of DEGs, with day 4 post infection providing the highest number of DEGs (S10 File). The scoDEG overlap for mACE2-hACE2 (4 dpi) vs. human studies was 35% for up-regulated scoDEGs and 15% for down-regulated scoDEGs (Fig 6C). The same comparisons for K18-hACE2 Suhrbier (4 dpi) vs. human studies provided 21% and 23% (Fig 6D), and for K18-hACE2 Winkler (4 dpi) vs. human studies gave 33% and 27%, respectively (S9A Fig). Thus, despite the lower viral loads (Fig 6A) and the endogenous mACE2 promoter driving hACE2 expression, the poor scoDEG overlaps were retained for mACE2-hACE2 mice vs. human groups.

The overlap for up-regulated DEGs for 4 dpi mACE2-hACE2 vs. 4 dpi K18-hACE2 was high, 76% (S9B Fig), with both strains on a 6J background. The concordance for IPA Cytokine USRs for this comparison was also highly significant (S9C Fig). These data argue that the promoter that drives hACE2 expression does not play a major role in determining the nature of the inflammatory responses. The lower overlap in down-regulated DEGs (30%, S9B Fig) may be associated with the differences in viral loads (Fig 6A), but may also reflect infection of different cell types, given that hACE2 is driven by distinct promoters in these two models.

### Pathway comparisons between infected mACE2-hACE2 and human lungs

**Fig 7:**
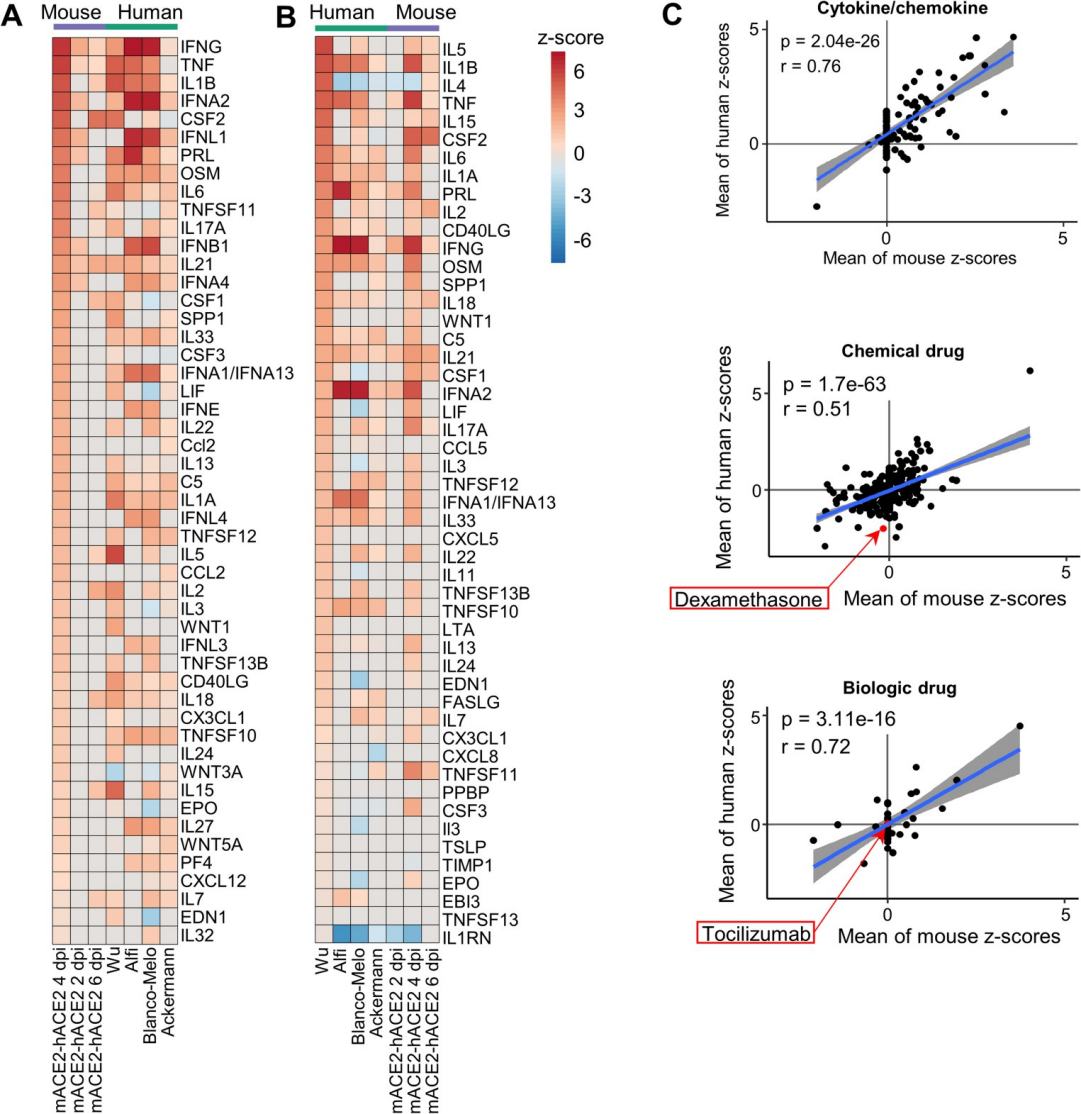
Cytokine/chemokine and drug USR concordances between mACE2-hACE2 mice and humans. **(A)** Heatmap comparing mACE2-hACE2 mouse groups with human groups for IPA cytokine/chemokine USRs ranked by activation z-score in mACE2-hACE2 4 dpi. **(B)** Heatmap comparing groups as in A, except ranked according to z-score in Wu, as a representative of human groups. **(C)** Pearson correlation of mean mouse vs. mean human z-scores for significantly enriched cytokine/chemokine USRs (n = 136), chemical drug USRs (n = 959) and biologic drug USRs (n = 94). Blue line shows linear regression with 95% confidence intervals (grey).

The number of DEGs obtained from mACE2-hACE2 infected lungs was substantially lower than that obtained from K18-hACE2 infected lungs (S10 File vs. S1 File). Nevertheless, when analyzed by IPA, inflammatory pathways again (as for K18-hACE2 mice, Fig 4A and 4C) showed a high level of concordance with human studies (Fig 7A and 7C). Significantly enriched Chemical drug USRs again included dexamethasone (Fig 7C, Chemical drug). Likewise, significantly enriched Biologic drug USRs again included tocilizumab (Fig 7C, Biologic drug).

### Dominant segregation is by species rather than batch

**Fig 8:**
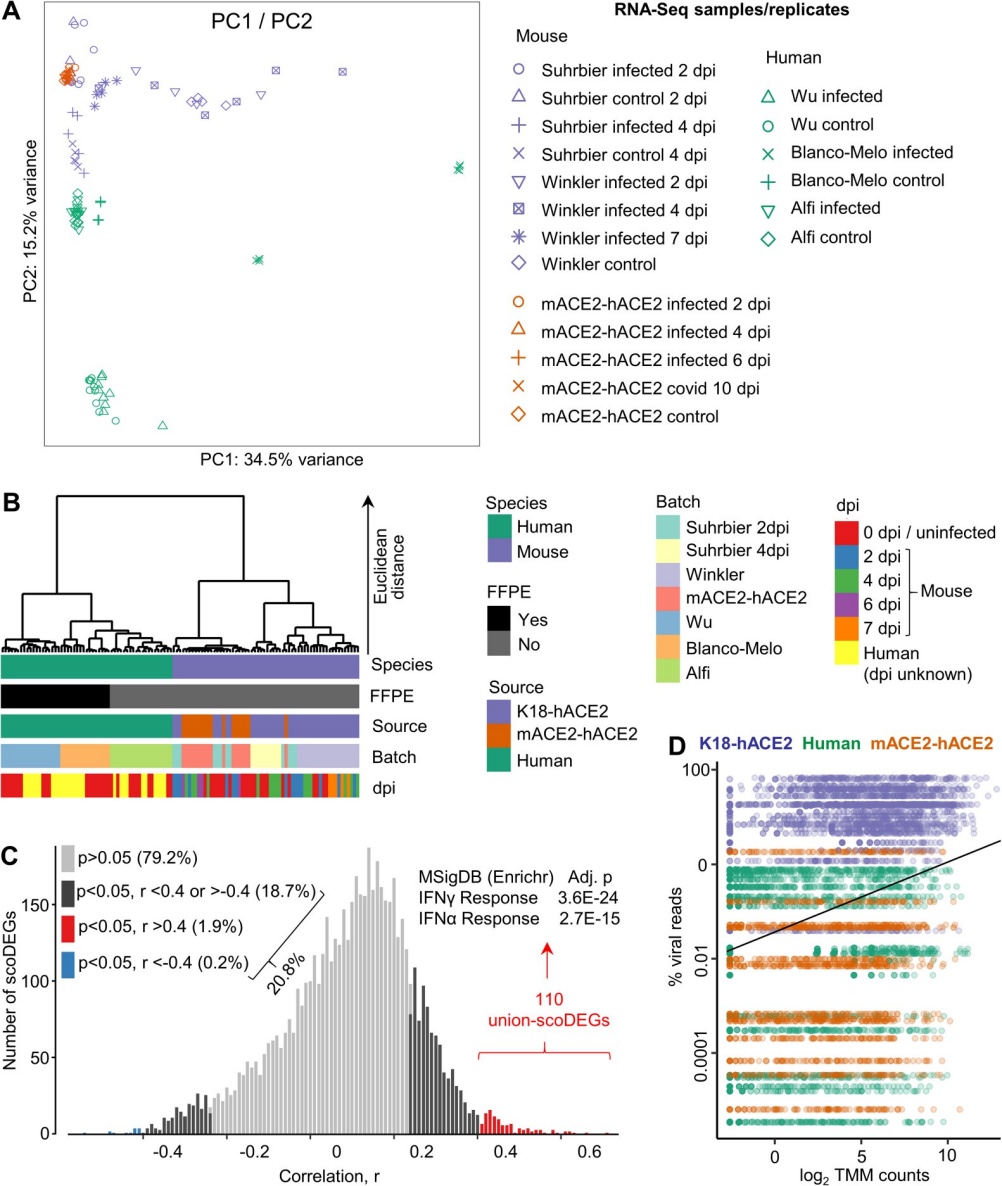
Principal component, hierarchical cluster and viral load analyses. **(A)** Scatter plots showing principal component 1 (PC1) vs. PC2 derived from 14,918 single-copy orthologues from all samples/accessions. Read counts were TMM-normalized and log_2_-transformed. Expression values were calculated by subtracting each TMM-log_2_ count from the row mean of all samples for each gene (i.e. deviation from row mean). **(B)** Hierarchical cluster analysis using the top 500 orthologues according to PC1 and PC2 loadings as in A. Clustering distance was Euclidean, and clustering method was Ward’s linkage. FFPE = Formalin-fixed, paraffin-embedded. (**C**) For all union-scoDEGs (a sco-DEG in at least 1 sample, n = 5880), across all samples/accessions (n = 69), Pearson correlations were undertaken comparing gene expression (log_2_ TMM normalized read count for each scoDEG) with percent viral reads (viral read count as a percentage of all read counts for host protein coding genes). Significance (p) and correlation (r) were generated for all scoDEGs. A histogram showing distribution of r values is shown, with colors indicating p and r cutoffs. The 110 genes that correlated well (red) were analyzed using the Molecular Signatures Data Base (MSigDB) available online via Enrichr, with the top 2 annotations shown. (**D**) The percent viral reads for the 69 samples/accessions are shown on the y axis, and were plotted against expression (log_2_ TMM counts) of the 110 genes in C. As expected, as correlating union-scoDEGs were selected from D (red), a significant correlation emerged when all 110 union-scoDEGs are taken together; linear regression (black line), p = 2.02 x 10E-149, r = 0.29.

The central message from the preceding analyses is that scoDEGs identified in mouse and human studies show a poor level of overlap. Arguably, this could be largely due to batch effects, with associated differences in sample treatment and days post infection (unknown for most human studies), rather than representing an effect primarily associated with species. To investigate the effect of species, batch, days post infection and viral reads, read counts from all RNA-Seq datasets were combined into a single count matrix containing 17,682 orthologues for all samples and replicates. Read counts were trimmed mean of M values (TMM) normalized and log_2_ transformed. A principal components analysis (PCA) is shown for all samples and illustrated a clear segregation between human and mouse data sets (Fig 8A). Although, source, batch and days post infection contribute to variance, hierarchical clustering using the top 500 genes according to PC1 and PC2 loadings, again showed a dominant segregation by species (Fig 8B). The Wu and Blanco-Melo samples were formalin-fixed and paraffin-embedded (FFPE), which is known to reduce RNA yield, increase RNA degradation, and influence the results of gene expression analyses [59–61]. However, although the read-depth of Blanco-Melo infected samples was low, read-depth of uninfected samples was comparable with that of non-FFPE groups (S1 Fig). In addition, Wu had the highest read-depth of any group. Importantly, clustering due to FFPE was not evident in the human clade of the hierarchical clustering analysis, in which Blanco-Melo (FFPE) (Fig 8B, Batch, orange) clustered with Alfi (not FFPE) (Fig 8B, Batch, green) rather than Wu (FFPE) (Fig 8B, Batch, blue). Although the day post infection is not known for human samples, the Euclidean distances separating uninfected human controls vs. infected human samples (irrespective of dpi) (Fig 8B, Human clade; red vs. yellow) were substantially less than those separating species (Fig 8B, Species, green vs. purple). Furthermore, clustering by dpi for mouse was also largely not evident (Fig 8B, Mouse vs dpi). Overall, these data argue the discordance described herein between mouse and human responses to SARS-CoV-2 infection in lungs is primarily due to differences in species, rather than due to batch effects, dpi or sample treatment.

### Poor scoDEG overlap between species does not associate with viral load

There were large differences in viral loads between K18-hACE2 and human groups (Fig 2A), which might explain, at least in part, the poor overlap in scoDEGs (Fig 1B). To further analyze the role of viral load, the number of union-scoDEGs (a scoDEG in at least 1 sample) whose expression correlated with viral load was determined across all infected samples/accessions. For each of the 69 infected samples (human n = 27, K18-hACE2 n = 27, mACE2-hACE2 n = 15), the “log_2_ TMM normalized read count for each scoDEG (RSEM)” and “the viral read count as a percentage of all read counts for host protein coding genes (RSEM)” was obtained. A Pearson correlation was then undertaken using the 69 data points, providing significance (p) and r for each of the 5880 union-scoDEGs. At a cut-off of p<0.05, expression of 20.8% of union-scoDEGs (1166 of 5880) correlated with viral load (Fig 8C, black, red and blue). At a cut-off of p<0.05 and r >0.4, only 1.9% of union-scoDEGs (110 of 5880) correlated with viral load (Fig 8C, red). As might be expected the latter union-scoDEGs were primarily associated with interferon (IFN) responses (Fig 8C, MSigDB). Using Spearman correlations these percentages were 47% (p<0.05) and 20% (p<0.05, rho>0.4), respectively. Thus expression of 20.8% of union-scoDEGs showed a significant linear correlation, and 47% a significant rank correlation, with viral load. The poor scoDEG overlap between K18-hACE2 mice and humans may thus, in part, be explained by the differences in viral loads.

When the percent viral reads are plotted for each of the 69 samples, the clear segregation between K18-hACE2 mice and human is again apparent (Fig 8D, y axis, purple vs. green), reproducing the data shown in Fig 2A. However, there was no segregation between percent viral reads for mACE2-hACE2 mice and human samples (Fig 8D, y axis, green vs. orange). That mACE2-hACE2 mice had much lower viral loads than K18-hACE2 mice was also shown by viral titrations (Fig 6A). Importantly, the poor mouse-human scoDEG overlap was largely retained for mACE2-hACE2 mice (Fig 6C), arguing that viral load differences are not a key determinant of the poor scoDEG overlap between species.

The x axis in Fig 8D shows the expression levels of the 110 union-scoDEGs from Fig 8C, illustrating that genes showing correlation between expression and viral load (at p<0.05, r>0.4) had a wide range of expression values (log_2_ TMM counts), rather than, for instance, being largely associated with low expressing genes. On average for these genes, a ≈10 fold increase in viral load increased expression by ≈30 fold (Fig 8D, black line).

### Poor scoDEG overlap is not dependent on transgenic expression of hACE2

An alternative to using hACE2 transgenic mice is the generation of mouse-adapted viruses that can utilize mACE2, allowing infection of wild-type mice [62–68]. To compare the gene expression profile induced by mouse-adapted SARS-CoV-2 to that observed in humans, we analyzed previously published data derived from C57BL/6J mice infected with the mouse-adapted SARS-CoV-2 strain, MA1 [68] (Table 1). Using an identical analysis pipeline to that used in the current study, Yan et al. reported 1027 DEGs due to MA1 infection at 4 dpi, of which 825 were scoDEGs. The scoDEG overlap for MA1 infection at 4 dpi vs. human studies was 26% for up-regulated scoDEGs and 19% for down-regulated scoDEGs (S10A Fig). These percentages are highly comparable with 35% and 15% for mACE2-hACE2, and 21% and 23% for K18-hACE2 vs. human, respectively (Fig 6C and 6D). The poor scoDEG overlap between mice and human studies (Fig 6C and 6D) was thus not restricted to mice with transgenic expression of hACE2.

When analyzed by IPA, a high level of concordance in pathways with human studies was again observed (as in Fig 7C) for MA1-infected mice (S10B Fig). Significant Chemical drug USRs again included dexamethasone (S10B Fig, Chemical drug), and significant Biologic drug USRs again included tocilizumab (S10B Fig, Biologic drug). Thus, the high level of pathway concordance (Fig 7C) was not reliant on transgenic expression of hACE2.

### Comparisons with acute lung injury models

**Fig 9:**
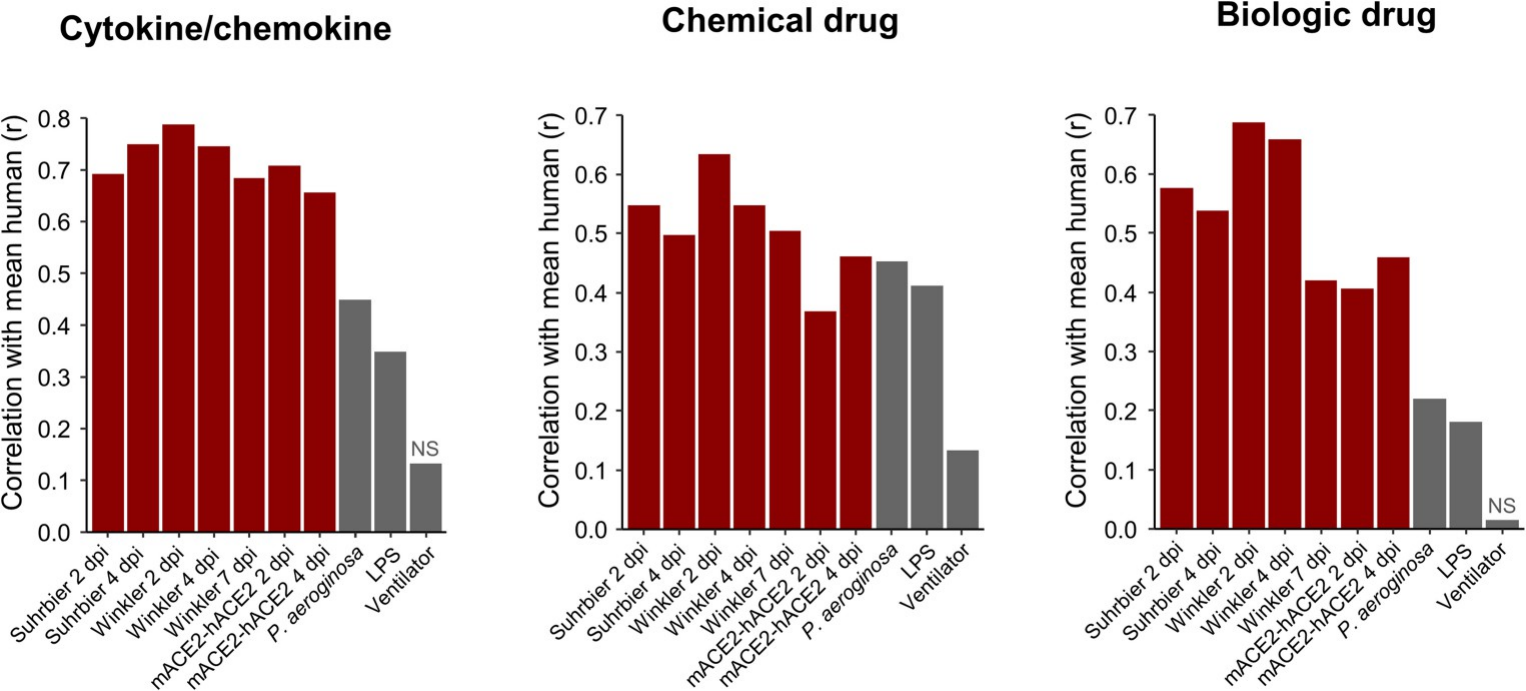
Cytokine, chemical and biologic drug pathways from acute lung injury models correlate poorly with those from SARS-CoV-2 infected lungs. Three acute lung injury (ALI) mouse model data sets were obtained from the SRA. Reads were mapped to the GRCm38 reference genome, counted, normalized, and analyzed with IPA in the same manner as for SARS-CoV-2 mouse and human groups. Z-scores for IPA USRs for Cytokine/chemokine (n = 127), Chemical drug (n = 794), and Biologic drug (n = 79) from each mouse group and the 3 ALI models were tested for their linear relationship with the mean USR z-scores of all human groups by Pearson’s correlation tests. ‘NS’ indicates non-significant correlations (p > 0.05).

To determine whether the signatures described herein simply reflect lung damage (given S6 File), we obtained three RNA-Seq datasets derived from experiments in which acute lung injury (ALI) was induced in wild-type mice via infection with *Pseudomonas aeroginosa*, administration of lipopolysaccharide (LPS), or mechanical ventilation (S11 File). Differential gene expression, IPA USR analyses and correlations with mean of human z-scores was performed as in Figs 4C and 7C. The correlations (r) for mouse vs. human SARS-CoV-2 infection (for IPA Cytokine, Chemical drug and Biologic drug USRs) were generally higher than for the mouse ALI groups vs. human SARS-CoV-2 infections, with the mechanical ventilation ALI model showing the lowest correlation (Fig 9). These results argue that SARS-CoV-2 infection pathway signatures described herein do not simply reflect a response to lung injury (S12 File).

## Discussion

We show herein that RNA-Seq studies from three mouse models of SARS-CoV-2 infection, the widely used severe K18-hACE2 model, the less severe mACE2-hACE2 model, and a model using mouse adapted virus (MA1) in C57BL/6J wild-type mice. All three mouse models showed poor scoDEG overlap with four studies of infected human lung tissues. The overlaps ranged from 21–35% for up-regulated scoDEGs and 15–27% for down-regulated scoDEGs (Figs 1B, 6C, S9A and S10A). In contrast, concordance between mouse and human studies for inflammatory pathways and immune-related signatures was highly significant (Figs 2B, 3A, 3B, 4A and 4D, 7 and S10B). Thus, although mouse and human gene expression studies provide largely distinct DEG sets, the DEG sets from the two species, nevertheless, indicate activation of common inflammation/immune pathways. As dominant pathways in immunity and inflammation are the target of most COVID-19 interventions, these mouse models can be viewed as providing representative and pertinent models for pre-clinical assessments of new interventions.

The K18 promoter in K18-hACE2 mice drives receptor expression in cells that ordinarily would not express ACE2 [19]. The mACE2-hACE2 mice might be viewed as more physiologically relevant, in that receptor expression is restricted to cells where the mACE2 promoter is active. Brain infection was not detected in mACE2-hACE2 mice perhaps because neuroepithelial cells [69] do not express hACE2. However, given brain infection was reported in a model similar to our mACE-hACE2 mice [39], the CCID_50_ assay used herein, with a detection limit of ≈2 log_10_CCID_50_/g (Fig 6A), may simply be too insensitive to detect brain infection in these mice. Surprisingly, the lower levels of viral infection in the lungs of mACE2-hACE2 mice was not associated with lower overall hACE2 mRNA expression levels (Fig 6B). One might speculate that in K18-hACE2 mice, certain lung cells aberrantly express ACE2 and become productively infected [70, 71] leading to higher viral loads. Either way, the poor overlap in DEGs for K18-hACE2 groups and human studies was not demonstrably due to hACE2 expression being driven by the non-physiological K18 promoter, as the poor overlap was largely retained for mACE2-hACE2 and C57BL/6J mice (Figs 6C and S10A). The cytokine/chemokine pathways identified in infected K18-hACE2, mACE2-hACE2, and C57BL/6J lungs were similar (Figs 4A and 7A [32]), arguing that the promoter is not a major factor in determining the nature of the inflammatory responses following SARS-CoV-2 infection.

There are clearly a number of limitations for this kind of analysis. Unavoidable is the issue of single copy orthologues, which comprised 63–77% of genes identified by RNA-Seq in lung tissues. This issue is less of a problem for pathway analyses when using programs such as IPA that accept both human and mouse gene nomenclatures. The different sources of tissues and the different technologies used to generate gene expression data (Table 1) likely add to non-biological variability. In addition, the Wu study included a number of medicated patients, with data for matching medicated mice not available. However, the PCA and hierarchical cluster analysis argue that the dominant source of variance is species, supporting the view that the poor human-mouse scoDEG overlap has a predominantly biological rather than technical basis. The large differences in viral loads may play a role in the poor DEG/scoDEG overlaps, particularly for human groups and down-regulated genes, respectively. However, analyses of K18-hACE2 (high viral load) and mACE2-hACE (lower viral loads) argued that the difference in viral loads was not a major determinant of the poor overlap in scoDEGs for mouse vs. human. To what extent the poor overlap arises from intrinsic mouse-human differences in transcriptional responses to SARS-CoV-2 infection cannot readily be determined, especially when one is reliant on disparate patient studies. In reality, attempts to validate mouse models using transcriptional profiles from patient cohorts will likely always be confronted with such limitations. In addition to providing bioinformatic processes that can be used to compare mouse and human responses, the conclusions from the current study were strengthened by analyzing four human studies and 3 mouse models, and by addressing a series of potential confounders.

In summary, the analyses herein argue that overlap in scoDEGs for lung tissues of mice and humans after SARS-CoV-2 infection is generally poor. In contrast, the concordance in immune and inflammation pathways was high, arguing that the mouse models provide relevant and pertinent models in which to evaluate new interventions for SARS-CoV-2 and COVID-19.

## Materials and methods

### Ethics statement

All mouse work was conducted in accordance with the “Australian code for the care and use of animals for scientific purposes” as defined by the National Health and Medical Research Council of Australia. Mouse work was approved by the Queensland Institute of Medical Research Berghofer Medical Research Institute’s (QIMR Berghofer MRI) animal ethics committee (P3600, A2003-607). For intrapulmonary inoculations via the intranasal route, mice were anesthetized using isoflurane. Mice were euthanized using CO_2_. All infectious SARS-CoV-2 work was conducted in a dedicated suite in a biosafety level-3 (PC3) facility at the QIMR Berghofer MRI (Australian Department of Agriculture, Water and the Environment certification Q2326 and Office of the Gene Technology Regulator certification 3445).

### Viruses and virus preparations

The SARS-CoV-2 isolate (hCoV-19/Australia/QLD02/2020) was kindly provided by Dr Alyssa Pyke and Fredrick Moore (Queensland Health Forensic & Scientific Services, Queensland Department of Health, Brisbane, Australia). The virus sequence is deposited at GISAID (https://www.gisaid.org/) [31]. Virus stocks were prepared in Vero E6 cells as described [31] and were checked for mycoplasma as described [72]. The fetal calf serum used for propagation of cells and virus was checked for endotoxin contamination as described [73].

The mouse adapted virus, MA1, has been described previously and was derived from the aforementioned virus (SARS-CoV-2_QLD02_) by *in vitro* passage and was used to infect C57BL/6J mice as above [32].

### K18-hACE2 mice

K18-hACE2^+/-^ mice were purchased from Jackson laboratories and were maintained in-house as heterozygotes by backcrossing to C57BL/6J mice [27,30]. Mice were typed as described [31] using hACE2 Primers: Forward: 5’-CTT GGT GAT ATG TGG GGT AGA -3’; Reverse: 5’-CGC TTC ATC TCC CAC CAC TT -3’ (recommended by NIOBIOHN, Osaka, Japan).

### mACE2-hACE2 mice

These mice were generated by Monash Genome Modification Platform (MGMP), Monash University and are freely available through Phenomics Australia (MGMP code ET26). Briefly, the mouse BAC clone RP23-152J15 was obtained from BACPAC Genomics and was used to generate a mACE2 promoter subclone. hACE2 cDNA with a polyA tail was cloned into mACE2 promoter subclone. The hACE2 (ENSG00000130234) sequence was codon optimized for mouse expression and was ordered as a synthetic cDNA with homology arms (GeneArt). The transgenic construct contained the mACE2 promoter and hACE2 followed by a poly A (S8A Fig). A maxiprep was then digested with Asc1 and the extracted 10,062 fragment (2.5 ng/ml) microinjected into the pronucleus of C57BL/6J zygotes at the pronuclei stage. Injected zygotes were transferred into the uterus of pseudo pregnant F1 females.

Mice were genotyped using the following primers 5’-TCC GGC TGA ACG ACA ACT CC -3’, 5’-TAT GTT TCA GGT TCA GGG GGA GG -3’. Cycling conditions were: 1 cycle at 94°C for 3 mins; 35 cycles of 94°C for 30 secs, 60°C for 30 secs, and 72°C for 1 min; and 1 cycle of 72°C for 10 min followed by cooling to 4°C. Fragments were run on a gel with a 374 bp band indicating the presence of the transgene (S8B Fig). The mACE2-hACE2 mouse line was maintained in-house as heterozygotes by backcrossing onto C57BL/6J mice.

### Mouse SARS-CoV-2 infections

Mice were infected intrapulmonary via the nasal route with 5×10^4^ CCID_50_ of virus in 50 μl medium while under light anesthesia; 3% isoflurane (Piramal Enterprises Ltd., Andhra Pradesh, India) delivered using The Stinger Rodent Anesthesia System (Advanced Anaesthesia Specialists/Darvall, Gladesville, NSW, Australia).

Mice were scored daily on a scale of 0–3 (Diseases scores) according to posture, activity, and fur ruffling. For all criteria, the normal condition was designated as 0. For posture, hunching only while at rest was designated as 1, moderate hunching with some impairment of normal movement was designated as 2, and severe hunching with difficulty in maintaining upright posture was designated as 3. For activity, a mild to moderate decrease was designated as 1, stationary unless stimulated was designated as 2, and reluctant to move even if stimulated was designated as 3. For fur ruffling, mild to moderate fur ruffling was designated as 1, severe ruffling was designated as 2, and shivering was designated as 3. Any animal reaching a level of 3 in any single criterion was euthanized, and any animal reaching a level of 2 in two or more criteria was euthanized.

Body weight was measured daily. Viral titrations were performed at 5 days post-infection with a CCID_50_ assay using Vero E6 cells and serial dilution of supernatants from homogenized tissues as described previously [31].

### Gene expression analysis

Suitable human and mouse COVID-19 transcriptome datasets were identified by searching the National Centre for Biotechnology Information Sequence Read Archive (NCBI-SRA) via the BigQuery platform using the command: ‘SELECT distinct m.bioproject FROM nih-sra-datastore.sra.metadata as m, UNNEST (m.attributes) as a WHERE (m.organism = ’Homo sapiens’ OR m.organism = ’Mus musculus’) AND assay_type = ’RNA-Seq’ AND ((a.v LIKE ’SARS%2’) OR (a.v LIKE ’COVID%’))’. The search was also extended to include micro-array data and non-publicly available data by searching the NCBI-PubMed database using search terms: ‘COVID’ and ‘SARS’. Microarray data relating to Ackermann et al. (2020) were accessed via the Vivli Centre for Global Clinical Research Data.

Raw sequence data for Winkler, Wu, Blanco-Melo and Alfi groups (see Table 1), and *P*. *aeruginosa*, LPS, and mechanical ventilation datasets (see S11 File), were accessed from SRA using fasterq-dump from SRA-toolkit. Raw sequence data for Suhrbier K18-mACE2 and hACE2-mACE2 were generated by for this study as described above. Quality control of fastq files was performed using FastQC v0.11.9 [74]. Adapter sequences were identified using BBmerge from the BBmap package v38.90 [75], and FastQC. Reads were trimmed to remove adapter content, size-selected to remove reads less than 36 nt in length, and quality-filtered to remove reads with less than a Q20 Phred score within a sliding-window tetramer, using Trimmomatic v0.36 [76]. Processed reads were aligned to either the GRCm39 vM26 or GRCh38 v37 reference genome for mouse and human datasets, respectively, using STAR aligner v2.7.1a [77]. Prior to alignment, each reference genome was augmented to include the NC_045512 SARS-Cov-2 Wuhan-Hu-1 viral genome. The number of reads mapping to SARS-CoV-2 was calculated using Samtools v1.9 [78]. For paired-end datasets, only primary proper pairs were counted. Host gene expression was calculated using RSEM v1.3.1 [79]. DEGs were identified using Bioconductor v3.13 [80] and EdgeR v3.34.0 [81] in R v4.1.0 [82]. Genes with read coverage of less than 2 counts per million were excluded from all further analyses. Following read-alignment, it was noted that the Blanco-Melo et al. data had very low sequencing depth in COVID-19 infected samples. Therefore, gene expression data were obtained from S6 Table of Blanco-Melo et al. [43]. A mouse-human orthologue table was extracted from the Ensembl database using BiomaRt v2.48.2 [83] in R. The table was used to filter DEGs to generate orthoDEG lists. A single copy orthologue DEG list (scoDEG) was generated by retaining only orthoDEGs for which there was only a single copy in each species.

Approximately 8% of human-mouse single-copy orthologues have gene IDs that differ between species. Prior to performing any cross-species comparison of scoDEGs, all mouse IDs were changed to human. Overlap between groups for DEGs and scoDEGs was calculated in R and plotted using Eulerr v6.1.0 [84] in R. Pearson’s correlation of log_2_ fold-changes using the union of scoDEGs for each pairwise combination of groups was calculated in R. In instances where data was missing for a scoDEG in one group (due to failing CPM >2 threshold for RNA-Seq data or being absent from the immune gene panel for Ackermann data), the scoDEG was excluded from that pairwise correlation. The proportion of up- and down-regulated DEGs and scoDEGs shared between groups was calculated in R and plotted using ggVennDiagram v1.1.4 [85] in R. Mean mouse log_2_ fold-change and mean human log_2_ fold-change were compared by Pearson’s correlation using scoDEGs that were significant in at least one human group and/or at least one mouse group. In instances where data was missing for a scoDEG in a particular group, log_2_ fold-change of that scoDEG was made zero for that group.

### Reciprocal gene set enrichment analysis

For each group, a log_2_ fold-change ranked gene list was produced using DESeq2 [86] with default settings. Also for each group, orthoDEG sets were filtered to retain only the top 50% of orthoDEGs when ranked according to absolute log_2_ fold change. (Removal of the bottom 50% of genes avoids including in the analysis genes with low fold change and genes ranked by very small differences in fold change). For all ranked gene lists and filtered orthoDEG sets, gene IDs of mouse-human orthologues were standardized by substituting mouse IDs for their human equivalent where gene IDs differed between species. A Gene Set Enrichment Analysis using GSEA v4.1.0 [45] with 100 permutations and the ‘no_collapse’ setting was used to test for enrichment of filtered orthoDEG sets within ranked gene lists.

### Immune-SigDB gene set enrichment analysis

For each group, log_2_ fold change ranked gene lists were produced as described above, except that no standardization was performed on gene IDs of mouse-human orthologues. The Immune-SigDB v7.4 [15] gene set collection comprising 5219 immune-related gene sets was obtained from the Molecular Signatures Database [87]. A gene set enrichment analysis was performed as described above to test for enrichment of Immune-SigDB gene sets within ranked gene lists. Any gene set not found to be significantly enriched in a particular ranked gene list was given a NES of zero for that group.

### Pathway analysis

Pathway analysis was performed using Ingenuity Pathway Analysis (IPA) v65367011 (Qiagen) with default settings. Data were plotted using pheatmap v1.0.12 [88] and ggplot2 v3.3.3 [89] in R. Gene networks were constructed using USR output and the My Pathways tool in IPA. For each of the IL-6R, TNF, and IFNg USRs, a list of ‘Molecules in dataset’ was obtained for each group. ‘Molecules in dataset’ from each group were then concatenated to create a single molecule list related to each of the three USRs of interest. Each molecule list was used as input to the My Pathways tool. Starting with each USR, the ‘Build/Grow’ function was used to identify direct and indirect downstream relationships between that USR and any of the molecules in its respective molecule list. The following parameters were set as follows: ‘Data Sources’ was set to all; ‘Confidence Level’ was set to ‘Experimentally observed’; ‘Species’ was set to human and mouse; all ‘Tissues and Cell Lines’ were selected except for those relating to cancer; all ‘Relationship Types’ were selected. To identify targets of transcription factors, a second round of ‘Build/Grow’ was performed in same manner as the first except that only direct relationships were allowed, and relationships had to include the transcription factors identified in the first round of ‘Build/Grow’. Each network was then exported in tabular format and plotted using Cytoscape v3.8.2 [90].

### *Nnt* genotyping

Mouse RNA-Seq data were interrogated for the presence of exon two and nine of the nicotinamide nucleotide transhydrogenase (*Nnt*) gene as described [58] using Repair and BBduk from the BBmap package v38.90. Reads containing at least one 31-mer exactly matching either exon were counted as belonging to that exon. If one member of a read pair matched an exon, the other member was also counted as a match.

### Statistics

Statistics were performed using IBM SPSS Statistics for Windows, version 19.0 and R version 4.1.0. For gene expression and pathway enrichment data a Pearson’s correlation test was used in accordance with the central limit theorem. The relationship between gene expression and viral load was tested using Pearson’s correlation and Spearman’s rank tests. For mouse data (weight change, disease scores, virus titers) the non-parametric Kolmogorov-Smirnov (differences in variance was >4, skewness < -2, and/or kurtosis was >2) or Mann Whitney U tests (differences in variance was <4, skewness < -2, and/or kurtosis was >2) were used. Survival was compared between two groups using a log rank test.

## Supporting information

S1 Fig Number of RNA-Seq reads aligned to protein-coding genes. For each sample, reads were aligned to either the mouse GRCm39 M26 or human GRCh38 v37 reference genome using STAR. Reads aligning to protein-coding genes were counted using RSEM. The total number of reads aligned to protein coding genes are shown for each sample. Due to low coverage in Blanco-Melo infected samples, read data were not re-analysed for this dataset. Instead, differential expression results were obtained from the original publication [43].
(PDF)

S2 Fig Pair-wise comparisons between groups of differential gene expression. Upper-left Euler diagrams show the amount of overlap between groups regarding DEGs (green and purple circles) and scoDEGs (red and blue circles) for all possible group-wise combinations. Green and red circles relate to row names, while purple and blue circles relate to column names. Size of circles indicates the number of DEGs/scoDEGs, as produced by EdgeR analysis or, in the case of Ackermann and Blanco-Melo, as obtained from the authors. Lower-right Each cell contains information pertaining to the group-wise comparison indicated by the row and column names. Overlap—for each pair-wise comparison between groups the number of scoDEGs that were common to both groups is shown as a percentage of the total number of scoDEGs in the comparison.–log p and r—for each pair-wise comparison, gene expression was compared using the union of scoDEGs for those groups (i.e. single-copy orthologues that were differentially expressed in one or both groups, and that were present in the gene lists for both groups). Pearson correlations were then performed using the log_2_ fold-changes (log2FC) of those single-copy orthologues to provide–log p and r values. Ackerman provides high r values as this analysis only evaluated expression of 249 inflammation genes (see Table 1). Cells are colored using scales on the right. For upper left and lower right, colored boarders indicate whether comparisons are mouse-human (green), human-human (blue), or mouse-mouse (orange).
(PDF)

S3 Fig DEG overlap among human and K18-hACE2 mouse groups (A) All human groups were compared for overlap of up- and down-regulated DEGs. ‘n’ refers to the number of DEGs for each group. Within each segment of each Venn diagram the percentage of DEGs exclusive to that group (i.e. a DEG in that group but no other group) is provided as a percentage of the total number of DEGs in that group (e.g. 9/20 x 100 = 45%). The boxed percentages (any overlap) refer to the percent of all DEGs in the Venn that are shared by at least 2 groups. (B) As for A, except comparing DEGs between all K18-hACE2 mouse groups.
(PDF)

S4 Fig Correlation of group z-score vs. mean z-score for comparing cytokine/chemokine USR activation between species. Activation z-scores for each group are plotted on x-axes (left column = mouse groups, right column = human groups). Mean z-scores for each species are plotted on y axes (left column = mean of all human groups, right column = mean of all mouse groups).
(PDF)

S5 Fig Differential expression of 1000 genes associated with TNF signaling. **(A)** Regulatory network for TNF signaling was constructed in the following manner: DEGs from each group were used as input for a separate IPA Core Analysis. The ‘Upstream Regulators’ output was used to identify genes associated with TNF signaling. Results from all groups were concatenated into a single list of 1000 genes. This list was used to interrogate DEG lists from each group in order to identify which TNF-associated genes were up-regulated in each group. Node colour indicates whether a gene was up-regulated in mouse only (>1 mouse group, and no human), human only (>1 human group, and no mouse), both (> 1 mouse and > 1 human group), or none. Large sub-networks are labeled according to their hub node. **(B)** Heatmap comparing groups according to log_2_ fold-change (log_2_FC) of 1000 genes associated with TNF signaling. Genes are ordered according to log_2_FC in Wu. **(C)** Heatmap comparing groups according to differential expression of 1000 genes associated with TNF signaling. Genes are ordered as in B. Cells are coloured according to whether the gene was significantly up-regulated (red), significantly downregulated (blue), or not significant (NS, grey). The number of TNF genes that were significantly differentially expressed is shown for each group as a percentage of the total number of DEGs for that group (n).
(PDF)

S6 Fig Differential expression of 862 genes associated with IFNg signaling. **(A)** Regulatory network for IFNg signaling was constructed in the following manner: DEGs from each group were used as input for a separate IPA Core Analysis. The ‘Upstream Regulators’ output was used to identify genes associated with IFNg signaling. Results from all groups were concatenated into a single list of 862 genes. This list was used to interrogate DEG lists from each group in order to identify which IFNg-associated genes were up-regulated in each group. Node colour indicates whether a gene was up-regulated in mouse only (>1 mouse group, and no human), human only (>1 human group, and no mouse), both (> 1 mouse and > 1 human group), or none. Large sub-networks are labeled according to their hub node. **(B)** Heatmap comparing groups according to log_2_ fold-change (log_2_FC) of 862 genes associated with IFNg signaling. Genes are ordered according to log_2_FC in Wu. **(C)** Heatmap comparing groups according to differential expression of 862 genes associated with IFNg signaling. Genes are ordered as in B. Cells are coloured according to whether the gene was significantly up-regulated (red), significantly downregulated (blue), or not significant (NS, grey). The number of IFNg genes that were significantly differentially expressed is shown for each group as a percentage of the total number of DEGs for that group (n).
(PDF)

S7 Fig Wu-exclusive up-regulated DEGs. **(A)** Overlap between all human groups for human-exclusive up-regulated DEGs (up-regulated in any human group, but no mouse group). 1298 genes were upregulated only in Wu and no other human or mouse groups. **(B)** Although only up-regulated in Wu these 1298 DEGs, nevertheless, return very similar IPA Cytokine USR pathways as those shown in Fig 4A and 4B. **(C)** The 3%, 18% and 21% of DEGs in the IL6R, TNF and IFNg networks (Fig 4D IL6R, S5 Fig TNF, and S6 Fig IFNG) that were up-regulated only in human (green) comprised 194 genes. Of these DEGs, 73% were found up-regulated exclusively in the Wu dataset. **(D)** When these 143 DEGs were analysed by IPA Diseases or Functions the highest and lowest annotation by z-score suggest more cell survival and less cell death, consistent with Fig 3C. Thus IL6R, TNF, and IFNG networks contain genes that are also associated with cell survival. The presence of DEGs in these later networks that are only up-regulated in humans (Fig 4D IL6R, S5 Fig TNF, and S6 Fig IFNG, green) is largely due to the Wu dataset. The RNA-Seq data suggests that the tissues used to generate the Wu dataset had less virus (Fig 2A) and less cell death (as also seen in Fig 3C), with pathways somewhat distinct (Fig 3A), perhaps because these samples were collected at a later time point when recovery was well underway and/or because a series of medication were used by the patients. The 3%, 18% and 21% of network genes up-regulated in humans might suggest humans up-regulate these network genes in response to SARS-CoV2 infection, whereas mice do not. However, this may largely be due to the fact that no comparable mouse data set was available (e.g. medicated in the same way).
(PDF)

S8 Fig mACE2-hACE2 mice. **(A)** The transgenic construct used for generation of mACE2-hACE2 mice containing the mACE2 promoter and hACE2 followed by a poly A. **(B)** Genotyping transgenic mice, a 374 bp PCR fragment indicates the presence of hACE2. **(C)** mACE2-hACE2 mice (n = 16 on day 0) were weighed at the indicated times, with 4 mice euthanized on days 2, 4, 6 and 10. K18-hACE2 mice were infected with the same dose of SARS-CoV-2_QLD02_ (n = 8) and were all euthanized on day 5. **(D)** Nasal turbinate tissue titers on the indicated days post infection. Limit of detection ≈2 log_10_CCID_50_/g (ND–ND detected). **(E)** Lung H&E 6 dpi showing loss of alveolar spaces (a—remaining spaces) (left), cellular infiltrates (white dashed ovals), smooth muscle hypertrophy/hyperplasia (h), and bronchial sloughing (black dashed oval).
(PDF)

S9 Fig **(A)** Venn-diagrams show overlap in up- and down-regulated scoDEGs between Winkler 4 dpi and four human groups. Boxed overlap percentages represent the overlap in scoDEGs between K18-hACE2 Winkler 4 dpi and any human study. **(B)** Venn-diagrams show overlap in up- and down-regulated DEGs between mACE2-hACE2 4 dpi and K18-hACE2 Suhrbier and Winkler groups on 2 and 4 dpi. Boxed overlap percentages represent the overlap in mACE2-hACE2 4 dpi DEGs and DEGs in any of the indicated K18-hACE2 groups. **(C)** Pearson’s correlation of z-scores for Cytokine USRs (n = 70) from IPA comparing 4 dpi from K18-hACE2 and mACE2-hACE2.
(PDF)

S10 Fig **(A)** Venn-diagrams show overlap in up- and down-regulated scoDEGs between mouse-adapted SARS-CoV-2 (MA1) infected C57BL/6J mice at 4 dpi, and four human groups. Boxed overlap percentages represent the overlap in scoDEGs between C57BL/6J 4 dpi and any human study. **(B)** Pearson’s correlation of z-scores for Cytokine (n = 131), Chemical drug (n = 923), and Biologic drug (n = 87) USRs from IPA. Each correlation is comparing C57BL/6J MA.1 4dpi and the mean of all human groups.
(PDF)

S1 File DEGs, orthoDEGs, and scoDEGs from K18-hACE2 mice and humans in response to SARS-CoV-2 infection. (XLSX)

S2 File Union and intersection of K18-hACE2 mouse and human DEGs. (XLSX)

S3 File Percentage of scoDEGs in each group that correlate with viral load. (XLSX)

S4 File RNA-Seq gene lists ranked by log_2_ fold-change. (XLSX)

S5 File Reciprocal Gene Set Enrichment Analysis. (XLSX)

S6 File Diseases and Functions annotations enriched in down-regulated DEG lists. (XLSX)

S7 File Upstream Regulator annotations enriched in DEG lists. (XLSX)

S8 File Interaction network for IL6-R, TNF, and IFNg pathways. (XLSX)

S9 File log_2_ fold-changes of genes associated with IL6-R, TNF, and IFNg pathways. (XLSX)

S10 File DEGs, orthoDEGs, and scoDEGs from mACE2-hACE2 mice in response to SARS-CoV-2 infection. (XLSX)

S11 File Information relating to three acute lung injury mouse model RNA-Seq datasets. (XLSX)

S12 File Pearson correlations of cytokine/chemokine, chemical drug, and biologic drug z-scores for each mouse group versus the mean of all human groups. (XLSX)
