# Supplementary material for: Mouse models of COVID-19 recapitulate inflammatory pathways rather than gene expression
Source: PLoS Pathog. 2022 Sep 26;18(9):e1010867. doi: 10.1371/journal.ppat.1010867 (PMC9536645; doi:10.1371/journal.ppat.1010867)
Supplement: S7 Fig — (A) Overlap between all human groups for human-exclusive up-regulated DEGs (up-regulated in any human group, but no mouse group). 1298 genes were upregulated only in Wu and no other human or mouse groups. (B) Although only up-regulated in Wu these 1298 DEGs, nevertheless, return very similar IPA Cytokine USR pathways as those shown in Fig 4A and 4B. (C) The 3%, 18% and 21% of DEGs in the IL6R, TNF and IFNg networks (Fig 4D IL6R, S5 Fig TNF, and S6 Fig IFNG) that were up-regulated only in human (green) comprised 194 genes. Of these DEGs, 73% were found up-regulated exclusively in the Wu dataset. (D) When these 143 DEGs were analysed by IPA Diseases or Functions the highest and lowest annotation by z-score suggest more cell survival and less cell death, consistent with Fig 3C. Thus IL6R, TNF, and IFNG networks contain genes that are also associated with cell survival. The presence of DEGs in these later networks that are only up-regulated in humans (Fig 4D IL6R, S5 Fig TNF, and S6 Fig IFNG, green) is largely due to the Wu dataset. The RNA-Seq data suggests that the tissues used to generate the Wu dataset had less virus (Fig 2A) and less cell death (as also seen in Fig 3C), with pathways somewhat distinct (Fig 3A), perhaps because these samples were collected at a later time point when recovery was well underway and/or because a series of medication were used by the patients. The 3%, 18% and 21% of network genes up-regulated in humans might suggest humans up-regulate these network genes in response to SARS-CoV2 infection, whereas mice do not. However, this may largely be due to the fact that no comparable mouse data set was available (e.g. medicated in the same way). (PDF) [file ppat.1010867.s007.pdf]

A

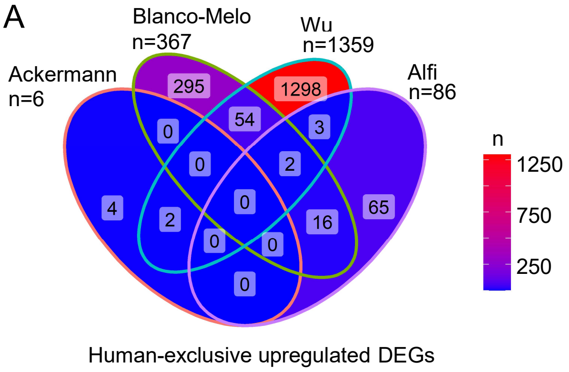

B

Upstream Regulator	Molecule Type	Activation z-score	p-value of overlap
IL4	cytokine	7.563	0.0441
IL5	cytokine	5.885	1.49E-08
CD40LG	cytokine	4.145	0.0157
TNF	cytokine	4.022	0.0252
IL15	cytokine	3.671	7.43E-05
IL1B	cytokine	3.483	0.0186
WNT1	cytokine	2.942	0.0415
IL2	cytokine	2.759	4.12E-05
IL3	cytokine	2.557	3.4E-09
EPO	cytokine	2.394	3.06E-06
OSM	cytokine	2.26	0.0235

C

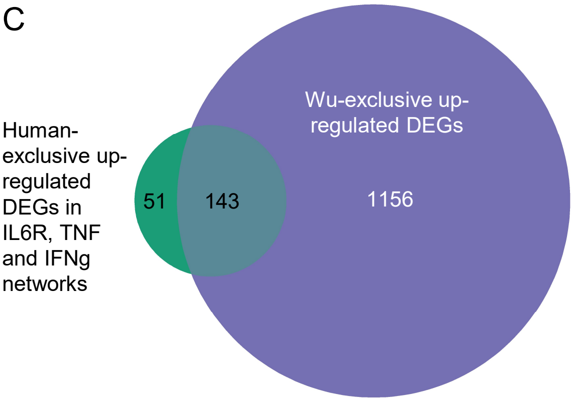

D

Diseases or Functions Annotation	p-value	Activation z-score
Cell survival	1.66E-23	5.32
Organismal death	6.18E-22	-7.348
